# Polymorphisms in the selectin gene cluster are associated with fertility and survival time in a population of Holstein Friesian cows

**DOI:** 10.1371/journal.pone.0175555

**Published:** 2017-04-18

**Authors:** Xing Chen, Shujun Zhang, Zhangrui Cheng, Jessica S. Cooke, Dirk Werling, D. Claire Wathes, Geoffrey E. Pollott

**Affiliations:** 1 Key Laboratory of Agricultural Animal Genetics, Breeding, and Reproduction of Ministry of Education, Huazhong Agricultural University, Wuhan, China; 2 Royal Veterinary College, North Mymms, Hatfield, Hertfordshire, United Kingdom; University of Florida, UNITED STATES

## Abstract

Selectins are adhesion molecules, which mediate attachment between leucocytes and endothelium. They aid extravasation of leucocytes from blood into inflamed tissue during the mammary gland’s response to infection. Selectins are also involved in attachment of the conceptus to the endometrium and subsequent placental development. Poor fertility and udder health are major causes for culling dairy cows. The three identified bovine selectin genes *SELP*, *SELL* and *SELE* are located in a gene cluster. *SELP* is the most polymorphic of these genes. Several SNP in *SELP* and *SELE* are associated with human vascular disease, while *SELP* SNP rs6127 has been associated with recurrent pregnancy loss in women. This study describes the results of a gene association study for SNP in *SELP* (n = 5), *SELL* (n = 2) and *SELE* (n = 1) with fertility, milk production and longevity traits in a population of 337 Holstein Friesian dairy cows. Blood samples for PCR-RFLP were collected at 6 months of age and animals were monitored until either culling or 2,340 days from birth. Three SNP in SELP_Ex4-6_ formed a haplotype block containing a Glu/Ala substitution at rs42312260. This region was associated with poor fertility and reduced survival times. SELP_Ex8_ (rs378218397) coded for a Val475Met variant locus in the linking region between consensus repeats 4 and 5, which may influence glycosylation. The synonymous SNP rs110045112 in SELE_Ex14_ deviated from Hardy Weinberg equilibrium. For both this SNP and rs378218397 there were too few AA homozygotes present in the population and AG heterozygotes had significantly worse fertility than GG homozygotes. Small changes in milk production associated with some SNP could not account for the reduced fertility and only SELP_Ex6_ showed any association with somatic cell count. These results suggest that polymorphisms in *SELP* and *SELE* are associated with the likelihood of successful pregnancy, potentially through compromised implantation and placental development.

## Introduction

Failure to conceive in a timely fashion is the major reason for culling dairy cows [1, 2]. Early and late embryo mortality and abortion are estimated to occur in around 40%, 20% and 5% respectively of all pregnancies in high yielding dairy cows [3]. The causes of these losses are diverse and include disease and metabolic imbalance [4]. There is, however, also clear evidence for a genetic component [5, 6]. Similar to the bovine situation, it has been estimated that 70% of conceptions in the human population are lost between fertilization and a live birth due to implantation failure, early pregnancy loss or abortion [7]. In humans, gross chromosomal abnormalities appear relatively more common [7, 8], whereas in cows advances in genome sequencing technologies have revealed a large number of loss-of-function variants, which are lethal during embryonic development [9].

Selectins are adhesion molecules with an N-terminal Ca^2+^ dependent lectin domain, an epidermal growth factor (EGF)-like module, a series of tandem consensus repeats, a trans-membrane domain and a cytoplasmic tail [10]. The three bovine selectin genes (*SELP*, *SELL* and *SELE*, also known as *CD62P*, *L* and *E*) are located in a gene cluster on BTA 16. Selectins are best known as key mediators in the interactions between leukocytes with endothelial cells, other leucocytes and activated platelets, which initiate tethering, adhesion, migration, other signaling events during inflammation, and as a marker for memory T cells [11, 12]. Following activation by inflammatory agents, such as thrombin, histamine or complement, SELP is rapidly transported to the endothelial cell surface where it can interact with the common selectin ligand P-selectin glycoprotein ligand 1 (PSGL1 also known as CD162 or SELPLG) expressed on the surface of leukocytes [13]. SELL is also expressed on the surface of the circulating leukocytes where it facilitates their secondary tethering of an already rolling neutrophil, further slowing down its movement [14, 15]. SELL can also form a complex with PSGL1, triggering integrin activation [16]. Like SELP, SELE also mediates the adhesion of cells to the vascular lining. SELE is generally up-regulated on the surface of endothelial cells by cytokine stimulation at sites of inflammation although it is constitutively expressed in bone marrow and skin [17]. We showed previously using an *in vitro* system that pre-activation of cultured bovine aortic endothelial cells (BoAEC) with lipopolysaccharide (LPS) caused a rapid (10 min) and slower (4 h) enhancement of adhesion by bovine peripheral blood leukocytes. This adhesion was accompanied by an up-regulation of both *SELP* and *SELE* mRNA expression in BoAEC and could be blocked using antibody against SELP [18].

In addition to fertility problems, the inflammatory disease mastitis is the second major cause of culling in dairy cows [2]. The selectin gene cluster is located close to a putative mastitis-related quantitative trait locus (QTL) [19–21]. Indeed, *SELL* is up-regulated on circulating neutrophils following bacterial infection and is important in causing their rapid influx into the udder [22]. Both, *SELL* and SELP were also up-regulated in bovine mammary tissue following experimental infection with either *S*. *uberis* or *E*. *coli* [23, 24].

Due to the strong interest in selectins as mediators of inflammation and markers for memory cells, a large number of studies in human patients have investigated associations between SNP polymorphisms in these genes with a variety of diseases. The main focus of these studies has been on cardiovascular disease, providing evidence that *SELE* polymorphisms are associated with an increased risk of myocardial infarction [25], coronary artery disease [26, 27] and essential hypertension [28], while SELP polymorphisms are associated with the risk of developing atherosclerosis [29], pulmonary hypertension [30] and venous thromboembolism [31]. These polymorphisms were also shown to explain around 5–15% of the variations in soluble SELP [29–32], SELL [33] or SELE [34] in circulation.

In the context of reproduction, one study has suggested that the *SELP* polymorphism rs6127 is associated with recurrent pregnancy losses in women [35]. This is of interest as selectins also play a role in both the initial adhesion between the embryonic trophoblast and the epithelial lining of the endometrium and the subsequent development of the placenta. In women *SELL*, *SELP* and *SELE* are all expressed in decidua, cytotrophoblast and syncitiotrophoblast [36]. SELL is present on the blastocyst surface and selectin oligosaccharide-based ligands are up-regulated on endometrium during the window of receptivity, playing an important role in the initial attachment phase [37, 38]. Women whose endometrial biopsies were low or negative for SELL ligand expression (tested using the monoclonal antibodies MECA-79 and HECA-452) were less likely to conceive [39–41].

A similar attachment system may be important in the cow as the SELL ligand podocalyxin and *PSGL1* were both expressed in the pre-implantation cow conceptus, while *SELL* and *SELP* (but not *SELE*) were up-regulated in the uterine endometrium between days 17–20 after insemination [42]. Knock-out of the *SELL* gene or SELL-ligand does not interfere with implantation in mice [43]. In the murine pregnancy, however, there is a tightly controlled recruitment of inflammatory cells to the feto-maternal interface to establish a population of leucocytes which are involved in regulating trophoblast invasion and local immunity: adhesion molecules are key to this process [44]. An excessive up-regulation of pro-inflammatory cytokines can trigger abortion in mice and both SELP and PSGL1 have been implicated [45, 46]. SELP is also up-regulated in decidual endothelial cells of women suffering spontaneous abortion [47]. These various studies suggest that polymorphisms in selectin genes could potentially regulate fertility through changes to the initial adhesion between conceptus and mother and/or by altering the later influx of inflammatory cells into the placenta, which may trigger abortion.

Analysis of the evolution of the selectin gene cluster in the human population found evidence for high nucleotide diversity in SELP exons 11–13 [48]. In particular, the haplotype frequency of the Val640Leu polymorphism (rs6133) differed between populations. Both this analysis and some of the disease association studies have provided evidence for a diversity in genotype frequencies for SNP in SELL and SELP according to European, Asian or African ethnicity [29, 32, 33]. In the cow, we previously investigated the presence of gene polymorphism for bovine selectins in a population of mainly British Holstein Friesian cows, together with several other dairy breeds (Friesian, Jersey, Ayrshire and Brown Swiss) [18]. *SELP* was highly polymorphic, with nine of the 13 SNPs identified located in its exons, whereas only three synonymous SNPs were found in *SELL* and one in *SELE*. The resulting amino acid changes for the three missense *SELP* SNP were located in the lectin domain and in two consensus repeat (CR) regions, CR2 and CR5. The Val475Met variant locus in the CR4 and CR5 linking region was very close to a predicted N-acetyl-D-glucosamine glycosylation site, which is likely to influence SELP function. The AA genotype of Val475Met was under-represented in the population, being found in only 1% of 373 heifers analyzed across the five dairy breeds, suggesting that AA homozygous embryos carrying this substitution may have compromised development.

In the present paper, we present results of a gene association study focused on the same population of Holstein Friesian cows for which extensive phenotypic information was available with regards to their fertility, milk production and disease from birth until culling [49, 50]. The aim was to determine whether polymorphisms in the three selectin genes were associated with infertility or mastitis and whether this in turn influenced the survival time of animals within the herds.

## Materials and methods

### Reagents and animals

All reagents were purchased from Sigma-Aldrich Company Ltd (Dorset, UK) unless otherwise stated. All procedures involving animals were carried out under the Home Office Animals (Scientific Procedures) Act 1986 and approved by the Royal Veterinary College’s (RVC) Ethics and Welfare Committee. Information on animals and farms has been described previously [49]. Briefly, samples and data from Holstein-Friesian heifers born between August 2003 and October 2004 on 18 commercial UK dairy farms and one primarily research farm were used for this study. Each farm had a four-month enrolment period during which consecutively born dairy heifers were recruited. The mean cohort size was 24 per farm, ranging from 15 to 30. These farms provided a variety of management practices representative of those commonly encountered on dairy farms in the UK. Whole blood samples for SNP identification (10 ml into heparinized vacutainers, BD Vacutainer System, Devon, UK) were collected from 336 Holstein heifers at 6 months of age and stored at -20°C.

### SNP identification in selectin transcripts (*SELP*, *SELL*, *SELE*)

The method used for SNP identification has been described previously [18]. DNA from blood samples was extracted using a FlexiGene DNA Kit (Qiagen, North Manchester, UK) following the protocol supplied and quantified with a NanoDrop ND-1000 spectrophotometer (NanoDrop Technologies Inc. Wilmington, USA). A DNA pooling sequence strategy was initially used to identify tSNPs in the exons of the three bovine selectin genes *SELP*, *SELL*, and *SELE* by direct sequencing of PCR amplification products. A total of 37 primers were designed using Primer Premier 5.0 software (Premier Biosoft, Palo Alto, CA, USA) based on the published gene sequences from the Ensembl database (ENSBTAG00000007307, ENSBTAG00000011515 and ENSBTAG00000020755 respectively), with NCBI Genbank accession numbers of NM_174181.2, NM_174182.1 and NM_174183.2, respectively. To construct a DNA pool, 1 μl of 50 ng μL^-1^ DNA samples from each of 10 British Holstein cows were pooled. PCR was performed using a Qiagen Multiplex PCR kit in a 40 μl reaction volume containing 50 ng DNA. The PCR reaction procedures were performed according to the manufacturer’s instructions, and resulting PCR products were sequenced by Source Bioscience (Nottingham, UK). Sequence files were assessed using the Chromas Pro software package (release 2.33; Technelysium Pty Ltd, South Brisbane, QLD 4101, Australia).

### Selectin genotyping by PCR-RFLP

Based on the results generated in the above pooling sequence analysis, five SNP from different exons in *SELP*, two in *SELL* and one in *SELE* were subsequently genotyped using PCR-RFLP. The appropriate enzymes were designed using the WatCut online tool (http://watcut.uwaterloo.ca/template.php?act=snp_new). Primers for PCR-RFLP were designed using Primer Premier 5.0. PCR was performed using a Qiagen multiplex PCR kit and the PCR reaction procedures were performed according to the manufacturer’s instructions. Thereafter, 10 μl PCR products were digested with 2 U restriction enzyme (New England Biolabs Inc, Hitchin, Herts, UK) in a 20 μl reaction volume at an appropriate temperature for each enzyme according to the instructions supplied. The digested products were detected by electrophoresis with 1.5% agarose gel. Alleles were indicated by bands of different sizes. The primers and restriction enzymes used for genotyping of SNP by PCR-RFLP are given in S1 Table and the genotypes of each cow are provided in S2 Table.

### Phenotypes

Data were obtained via regular visits to the farms and through their herd management software. The number of records used in each analysis varied depending on the availability of information on the genotype for each SNP and trait and the longevity of the individual cow. Fertility traits included the age in days at first and second calving (AFC and ASC), the interval to conception in the first and second lactation (d) and the calving intervals between the first and second and second and third lactations (d). The raw fertility phenotype data are given in S3 Table. Animals were recruited onto the study at birth and were monitored until 2,340 d (6.4 yr) or until the time of death or culling if that occurred earlier. The ages at culling are given in S4 Table. Reasons for culling were obtained where possible and classified into the following categories: infertility, abortion, mastitis/high somatic cell count (SCC), legs/feet/lameness, other illnesses (e.g. abscess, displaced abomasum), or no reason for culling given. Some animals were in more than one category, for example having a combination of poor fertility and high SCC [50].

Milk production traits were analysed for each animal in each of the first two lactations by measuring the total milk produced, 305 d milk yield and peak daily milk yield. These values were obtained from monthly records provided by commercial milk recording services. Data on SCC were available from monthly milk analysis records, which included this as one of the variables measured on the milk samples. Milk phenotype data are provided in S5 Table. Previous authors have used SCC data in a variety of ways to characterise the mastitis status of dairy cows. In these analyses a tertiary trait was derived from these data to represent affected, intermediate and unaffected animals after Yoshida et al. [51]. Unaffected animals never had a SCC >200,000 cells mL^-1^ (class 0). Affected cows, classified as mastitic, had a SCC >300,000 cells mL^-1^ in two consecutive tests or in four non-consecutive tests, or were cows with a SCC >500,000 cells mL^-1^ in any one test during any lactation period, regardless of parity (class 2). Intermediate animals were placed in class 1.

### Statistical analysis

The genotype frequencies of each polymorphism were examined for deviations from Hardy-Weinberg equilibrium within the population using the χ^2^ test. The extent of linkage disequilibrium between pairwise genotype combinations was also determined by calculating the correlation coefficient (r^2^) in Haploview software [52]. Association analyses were carried out for all traits except SCC, separately in each lactation using the mixed effect model in Eq 1.

$$Y_{ijkl}=\mu +H_{i}+{SNP}_{j}+a_{k}+e_{ijkl}$$ (1)

With: Y = trait analyzed; μ = overall mean; H_i_ = fixed effect of the ith herd (i = 1–20); SNP_j_ = fixed effect of the jth genotype of the SNP (j = 1–3), a_k_ = the random animal term fitted using a pedigree file and e_ijkl_ = random residual error.

For each cow, pedigree information for the preceding three generations was collected from the Holstein UK website (www.ukcows.com), creating a total pedigree containing 2,251 animals. There were 93 (± 6.0) sires (5.4 sires per heifer, range = 1–32) and 181 (± 1.8) maternal grandsires (2.0 maternal grandsires per heifer, range = 1–11). Significant differences between the genotypes within a SNP were analysed using the Students *t* test and standard errors of differences were calculated for each pair of means tested. Somatic cell count SCC was analysed using a trinomial generalized linear model with a logit link function. All analyses were carried out in ASReml [53]. The fertility traits analyzed in this study were transformed to their Log_e_ values where necessary to normalize their distribution.

Differences between genotypes in the number of animals that survived for each SNP were measured by Kaplan-Meier survival analysis using the Statistical Package for the Social Sciences 17.0 (SPSS Inc., Chicago, IL, USA) as described previously [54]. Censored animals were those cows that survived > 2,340 d (6.4 years). The proportions of cows censored were compared using the Cox proportional hazards regression model. The fixed effects of the herd and sire of the animal were initially included in the analyses but were found to have a non-significant effect on survival (*P* > 0.20) so were removed from the final model.

## Results

### Polymorphism of the bovine selectin genes (*SELP*, *SELL*, *SELE*)

As described previously [18], we initially identified 13 SNP in the exons of three selectin genes of a defined group of British Holstein Friesian cows. *SELP* was found to have the highest level of polymorphism as nine of the 13 SNPs were located in *SELP* exons, including three missense mutations. Three synonymous SNP existed in *SELL* and only one synonymous SNP in *SELE*.

Of these, five SNP from different exons in *SELP*, two in *SELL* and one in *SELE* were taken forward to genotyping in 337 Holstein Friesian heifers using PCR-RFLP. These SNP are described in Table 1.

**Table 1:** Identified polymorphisms of bovine selectin genes on BTA16 used in the genotype association study.

Gene	dbSNP ID	Alleles	Exon	SNP position (bp)	SNP type	Amino acid change
*SELP*	rs110033243	C/T	4	38,075,512	Synonymous	
	rs42312260	A/C	5	38,074,580	Missense	Glu/Ala
	rs137027551	A/G	6	38,068,897	Synonymous	
	rs378218397	A/G	8	38,057,153	Missense	Val/Met
	rs211179622	A/T	13	38,049,712	3’UTR	
*SELL*	rs109966956	A/G	3	38,170,687	Synonymous	
	rs41803917	C/T	4	38,168,866	Synonymous	
*SELE*	rs110045112	C/G	14	38,184,844	Synonymous	

### Genotype frequencies and linkage disequilibrium analysis

**Fig 1:**
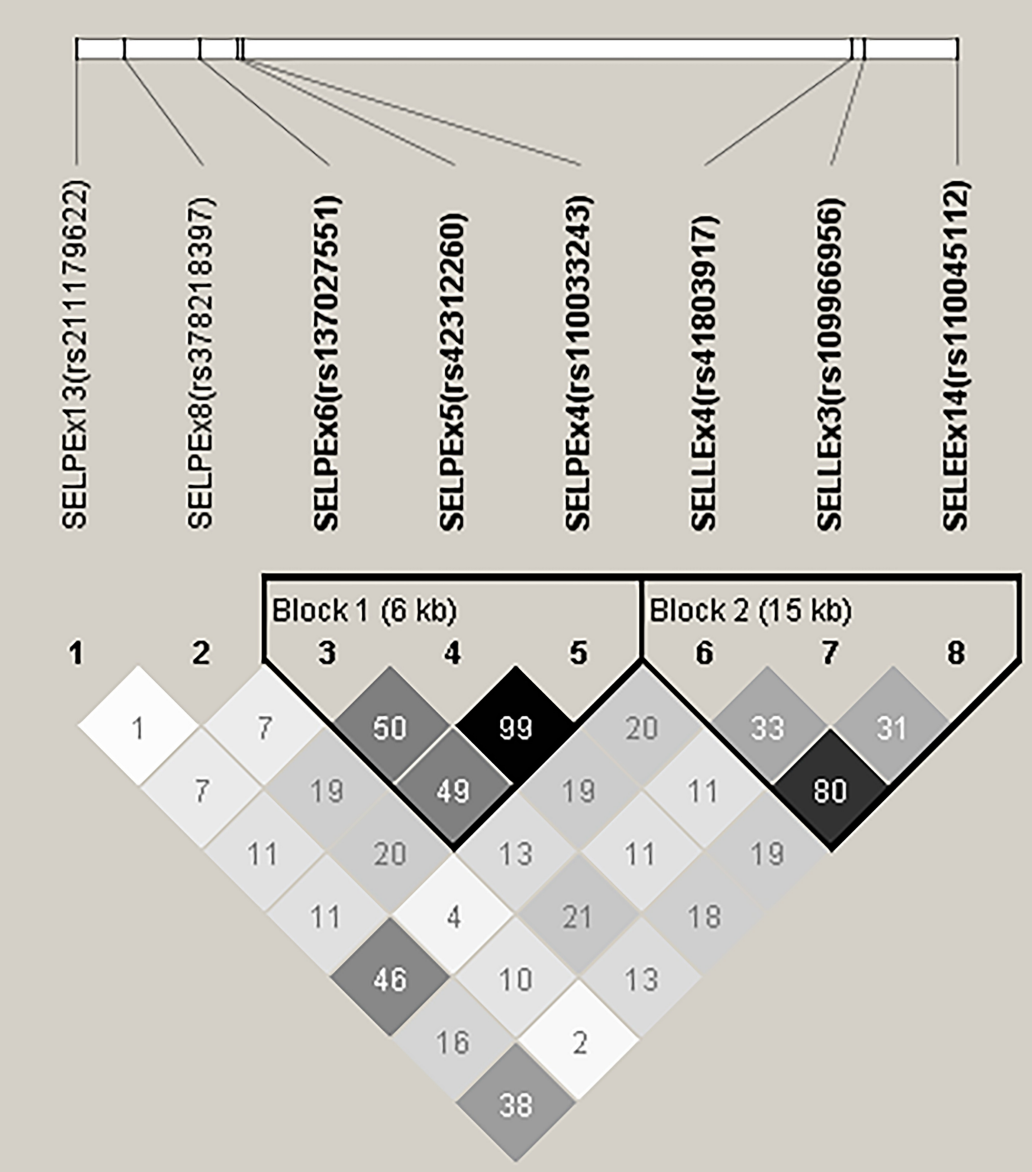
Linkage disequilibrium map for SNP in bovine selectin genes. Each SNP is represented by a grey triangle labelled 1 to 8, with their location on the chromosome shown above. The square in which the columns leading from two SNP intersect shows the correlation between them (r^2^ x 100), with darker shading representing higher correlation. The haplotype blocks represent SNP that are in high linkage disequilibrium with each other and thus are all generally inherited together.

A summary of allele and genotype frequencies for each SNP is shown in Table 2, along with the results of testing their distribution against the Hardy-Weinberg Equilibrium. For the *SELP* gene, SELP_Ex4_ and SELP_Ex5_ (rs110033243 and rs42312260) were in complete linkage disequilibrium (LD) with each other (r^2^ = 0.99) and formed a haplotype block with SELP_Ex6_ (Fig 1). The SNP rs378218397 and rs211179622 in SELP_Ex8_ and SELP_Ex13_ both tended to differ from HWE at P = 0.11. In both cases the heterozygotes and AA genotypes were under-represented. The one SNP rs110045112 in SELE_Ex14_ was also not in HWE (P < 0.03) as there were more heterozygotes and fewer GG homozygotes than expected. Linkage disequilibrium analysis showed that SELL_Ex4_, SELL_Ex3_ and SELE_Ex14_ were in another haplotype block.

**Table 2:** Genotype frequency of SNP in selectin genes in a population of Holstein cows.

					Genotype Number			Genotype frequency			HWE^a^
Gene	Exon	dbSNP ID	n	Alleles	Hom1^b^	Het	Hom2	Hom1	Het	Hom2	P
*SELP*	4	rs110033243	336	C/T	100	163	73	0.29	0.50	0.21	NS
	5	rs42312260	315	A/C	73	159	103	0.21	0.50	0.29	NS
	6	rs137027551	266	A/G	24	118	124	0.09	0.44	0.47	NS
	8	rs378218397	320	A/G	4	101	215	0.01	0.32	0.67	0.11
	13	rs211179622	331	A/T	11	131	189	0.03	0.40	0.57	0.11
*SELL*	3	rs109966956	330	A/G	97	158	75	0.29	0.48	0.23	NS
	4	rs41803917	317	C/T	181	118	18	0.57	0.37	0.06	NS
*SELE*	14	rs110045112	282	C/G	160	115	7	0.57	0.41	0.02	0.03

^a^HWE = Probability of χ^2^ to test if the genotypes are in Hardy-Weinberg Equilibrium

^b^Hom1, Het and Hom2 are the three SNP genotypes e.g. CC, CT, TT.

### Fertility and survival

**Fig 2:**
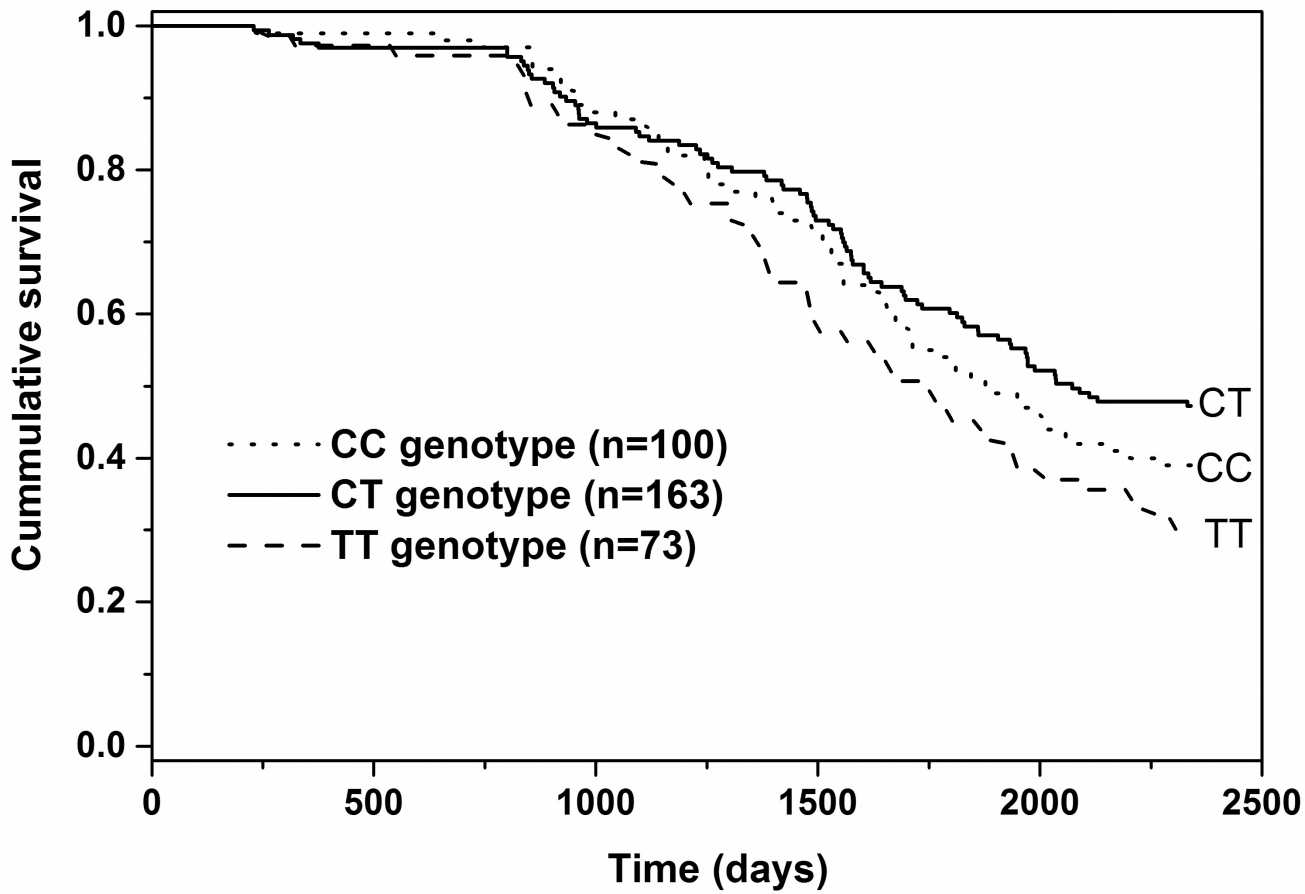
Kaplan—Meier analysis showing the proportion of animals from surviving from birth through to 2,340 d. A population of 336 Holstein Friesian cows were genotyped for the SELP_Ex4_ SNP rs110033243. Survival time was significantly lower for animals with the TT genotype (P < 0.05).

Significant associations between different SNP and fertility traits, outlined in Table 3, are summarised in Tables 4 and 5. SELP_Ex4_ and SELP_Ex5_ were both associated with days to conception in the second lactation, which was shorter by 12 to 19 days in the heterozygotes compared with the homozygotes. These SNP were also associated with survival (Fig 2). For SELP_Ex4_ the C allele was dominant. The survival time from birth to death/culling was 1,802, 1,850 and 1,691 d for the CC, CT and TT genotypes respectively. On average, the TT homozygotes therefore had lives that were about 4 months shorter than for animals carrying at least one C allele (P < 0.05). The survival curves showed that the genotypes did not start to diverge until after about 1,000 d of age (2.7 years), by which time most cows had started their first lactation. Within the TT genotype, 86% of cows reached first calving, similar to the 91% for each of the CC and CT genotypes. Only 45% of 73 TT cows reached their third lactation, however, compared with 57% and 60% for the CC and CT genotypes. These results indicated that the likelihood for culling increased in lactating animals. The reasons for culling were available for 41 animals with the SELP_Ex4_ TT genotype. The predominant reason was infertility, which accounted for 63% of the 41 culls, including five of the heifers, which failed to conceive at all and so never calved and six cows which aborted. Five of the infertile animals also had mastitis and a further five were culled due to mastitis alone, giving a total of 10/41 cows (26%) for which mastitis was a contributory reason for culling.

**Table 3:** Basic statistics of the traits analysed in this study.

Traits^a^	N	Mean	SD	Skewness^b^
Fertility Traits (d)				
AFC	310	812	123	1.3
DC_L1	272	128	90	2.4
CI_L1-2	253	413	99	1.8
ASC	253	1,223	159	0.9
DC_L2	207	122	72	1.6
CI_L2-3	185	403	76	1.4
No. calvings	337	2	2	-0.3
Milk traits (kg)				
TMY_L1	307	9,303	3,957	0.6
305d_L1	200	8,723	1,542	0.1
PY_L1	253	34	6	0.1
TMY_L2	237	10,109	3,144	-0.1
305d_L2	160	9,983	1,675	-0.2
PY_L2	206	42	7	-0.4

^a^L1, lactation 1; L2, lactation 2; AFC, age at first calving; ASC, age at second calving, DC, days from calving to conception; CI, calving interval; TMY, total milk yield; 305d, 305-day milk yield; PY, peak milk yield.

^b^Data were transformed to their Log_e_ values where necessary to normalize their distribution, as indicated by a skewness > 1 or < -1.

**Table 4:** Summary of results for milk and fertility trait probabilities (P < 0.1) associated with different selectin SNP in a population of Holstein cows^a^.

Gene	*SELP*					*SELL*		*SELE*
Exon	13	8	6	5	4	4	3	14
dbSNPID	rs211179622	rs378218397	rs137027551	rs42312260	rs110033243	rs41803917	rs109966956	rs110045112
AA change		Val/Met		Glu/Ala				
Fertility traits (d)			HapB1	HapB1	HapB1	HapB2	HapB2	HapB2
AFC		0.001***	0.046*					0.008**
DC_L1		0.029*	0.1					
CI L1-2			0.04*					
ASC		0.001***	0.001***					
DC_L2	0.08		0.07	0.045*	0.021*	0.01*	0.07	0.04*
CI L2-3	0.048*							
No. calvings		0.035*						0.058
Milk traits (kg)								
TMY-L1			0.004**		0.056			
								
								
PY-L1		0.095		0.095	0.089		0.042*	
305d-L1					0.09			
TMY-L2							0.091	
PY-L2			0.003**	0.004**	0.002**		0.041*	
305d-L2			0.075					0.036*

^a^ HapB, Haplotype block; L1, lactation 1; L2, lactation 2; AFC, age at first calving; ASC, age at second calving, DC, days from calving to conception; CI, calving interval; TMY, total milk yield; 305d, 305 day milk yield; PY, peak milk yield.

Significance is indicated as

*P<0.05

**P<0.01 and

***P<0.001.

**Table 5:** Associations between polymorphisms in selectin genes with fertility traits in a population of Holstein cows.

Gene/SNP	Fertility trait (d) #	Genotype			Mixed model residual SD	P §
*SELP*_*Ex4*_		CC	CT	TT		
rs110033243	n	65	104	38		
	DC_L2 log	4.76^a^	4.58^b^	4.71^a^	0.502	0.021
	DC_L2	117	98	111		
*SELP*_*Ex5*_		AA	AC	CC		
rs42312260	n	38	101	68		
	DC-L2 log	4.70^a^	4.59^b^	4.74^a^	0.504	0.045
	DC2	110	98	114		
*SELP*_*Ex6*_		GG	AG	AA		
rs137027551	n	115	108	23		
	AFC log	6.67^b^	6.71^a^	6.67	0.057	0.046
	AFC	788	820	788		
	n	87	97	19		
	CI_L1-2 log	5.97^b^	6.05^a^	5.99	0.021	0.04
	CI_L1-2	391	424	399		
	n	87	97	19		
	ASC log	7.07^b^	7.14^a^	7.09	0.092	0.001
	ASC	1176	1261			
*SELP*_*Ex8*_		GG	AG	AA		
rs378218397	n	199	92	3		
	AFC log	6.67^b^	6.73^a^	6.67	0.064	0.001
	AFC	788	837	788		
	n	180	76	2		
	DC_L1 log	4.64^b^	4.77^a^	5.46	0.051	0.03
	DC_L1	103	117	235		
	n	169	70	1		
	ASC log	7.08^b^	7.15^a^	7.31	0.09	0.001
	ASC	1187	1274	1495		
	n	215	101	4		
	No. calv.	2.66^a^	2.41^a^	1.0^b^	1.26	0.03
*SELP*_*Ex13*_		TT	AT	AA		
rs211179622	n	106	71	5		
	CI_L2-3 log	5.96^b^	6.02^a^	5.95	0.140	0.048
	CI_L2-3	387	412	384		
*SELL*_*Ex4*_		CC	CT	TT		
rs41803917	n	113	72	13		
	DC_L2 log	4.58^b^	4.78^a^	4.88^a^	0.514	0.01
	DC_L2	98	119	131		
*SELE*_*Ex14*_		CC	CG	GG		
rs110045112	n	103	68	3		
	DC_L2 log	4.59^b^	4.79^a^	4.87	0.516	0.04
	DC_L2	98	120	130		

# L1, lactation 1; L2, lactation 2; AFC, age at first calving; ASC, age at second calving, DC, days from calving to conception; CI, calving interval.

§ P = probability of a difference; within rows a > b (P < 0.05)

SELP_Ex6_, forming part of the same haplotype block as SELP_Ex4_ and SELP_Ex5_, was strongly associated with several fertility traits, with the AG heterozygotes having consistently worse fertility. Compared with the GG homozygotes they had a later age at both first and second calving, by 32 and 85 d respectively, and they also had a longer calving interval between the first and second lactations, by 33 d. This was in part because they required more services per conception in the first lactation (data not shown). Their survival times were, however, not affected.

SELP_Ex8_ was also negatively associated with fertility. This SNP had an unbalanced population distribution: only four animals were found in the population with an AA genotype and these survived fewer calvings (P < 0.05). Only half of the AA animals (2/4) calved at all, whereas for the AG and GG cows 91% and 90% respectively reached first calving. Subsequently the AG heterozygotes had significantly worse fertility than the GG homozygotes with older ages at both first and second calving by 49 and 87 d (P < 0.001) and a 14 d longer interval to conception in the first lactation (P < 0.05). For SELP_Ex13_ the AA genotype was also under-represented, although 9/11 animals did reach their first calving (82%). The heterozygotes performed worse than the TT homozygotes, tending towards a longer open period in the second lactation (P = 0.08) which resulted in a 25 d significantly longer calving interval between the first and second lactations (P < 0.05).

For SELL_Ex4_ the heterozygote CT cows had a longer interval to conception than CC by 21 d in the second lactation. The TT homozygotes were on average worse, but the difference did not achieve significance as only 13 animals were represented. The SNP in SELE_Ex14_ had an unbalanced distribution, with only 5/7 (71%) GG animals reaching first calving, compared with 89% CC and 93% CG genotypes, and they achieved fewer calvings in total (P = 0.058). The two GG animals which never calved were both culled for infertility as heifers. Subsequently the CG heterozygotes performed worse than the CC homozygotes, with a significantly longer open period by 22 d in the second lactation (P < 0.05).

### Milk yield and SCC

The basic statistics for the milk traits included in the analyses are shown in Table 3, with significant associations between different SNP and milk traits being summarised in Tables 4 and 6. For SELP_Ex4_ and SELP_Ex5_, heterozygote animals had a significantly higher peak milk yield (about 4 kg) in their second lactation. For SELP_Ex6_ the AG heterozygotes had longer first lactations by about 2 months than the GG genotype (377 v 313 d), resulting in higher total milk yields by 1,632 kg on average (10,154 v 8,522 kg). Their higher milk production continued into the second lactation in which the peak milk yields was significantly greater in the heterozygotes than in either of the homozygotes. SELP_Ex8_ and SELP_Ex13_ did not show any significant associations with milk yield in the first two lactations. For SELL_Ex3_ the GG homozygotes had higher peak yields than the AA homozygote by about 3 kg in each of lactations 1 and 2 but SELL_Ex4_ showed no significant associations. Finally, for the SNP detected in SELE_Ex14_, the CG heterozygotes were associated with a higher 305d milk yield in their second lactation (10,475 v 9,738 for the CC homozygote), a difference of 737 kg.

**Table 6:** Associations between polymorphisms in selectin genes with milk traits in a population of Holstein cows.

Gene/SNP	Milk trait (kg)#	Genotype			Mixed model residual SD	P§
*SELP*_*Ex4*_		CC	CT	TT		
rs110033243	n	61	88	42		
	PY-L2	40.8^b^	43.7^a^	41.2^b^	5.13	0.002
*SELP*_*Ex5*_		CC	AC	AA		
rs42312260	n	60	93	44		
	PY-L2	39.3^b^	43.6^a^	41.7 ^b^	5.87	0.004
*SELP*_*Ex6*_		GG	AG	AA		
rs137027551	n	114	107	23		
	TMY-L1	8522^b^	10154^a^	8753	3,601	0.004
	n	78	71	18		
	PY-L2	41.2^b^	44.0^a^	38.5^b^	5.40	0.003
*SELL*_*Ex3*_		AA	AG	GG		
rs109966956	n	67	122	60		
	PY-L1	32.4^b^	33.8^b^	35.2^a^	5.49	0.042
	n	50	104	49		
	PY-L2	39.7^b^	42.5^a^	43.0^a^	6.07	0.041
*SELE*_*Ex14*_		CC	CG	GG		
rs110045112	n	80	52	4		
	305d-L2 (kg)	9738^b^	10457^a^	9490	630	0.035

# L1, lactation 1; L2, lactation 2; TMY, total milk yield; 305d, 305-day milk yield; PY, peak milk yield.

§ P = probability of a difference; within rows a > b (P < 0.05)

The only SNP showing any significant effect of genotype on SCC was SELP_Ex6_ (P = 0.02). These results were based on a cumulative probability model starting with Class 0 (unaffected), 1 (intermediate) and 2 (mastitic). These are the three SCC classes that we used based on the previous report [51] to categorise the SCC results. For this the AA genotype had a higher proportion of healthy animals (32%) compared to AG and GG, which had no difference between them (22% and 18% respectively). The AA genotype cows also had a lower proportion of animals in the highest SCC category, classified as mastitic (18% compared with 32% AG and 27% GG). This suggested that the SELP_Ex6_ AA animals were more resistant to having a high SCC than the other two genotypes.

## Discussion

Selectins play a central role as adhesion molecules involved in leukocyte trafficking, giving them an important role in influencing inflammatory and immune responses [11]. These processes are crucial in maintaining health, but they can also lead to tissue damage if allowed to proceed unchecked [55]. Adhesion molecules are therefore likely to have experienced selection pressure during evolution [56]. An analysis of the selectin gene cluster showed that two regions in *SELP* and one in *PSGL1* showed high genetic differentiation in the human population. It was suggested that high nucleotide diversity in *SELP* exons 11–13 between Africans and Europeans might have been caused by natural selection responding to different environmental pressures [48]. This is highly likely to be driven by host-pathogen co-evolution, such as to malaria parasites, as SELP is involved in the adherence of *Plasmodium falciparum*-infected erythrocytes to vascular-endothelial cells [57]. Similar to the human system, we have shown previously that *SELP* is also highly polymorphic in the dairy cow population with a significant reversal in the breed distribution of SELP_Ex13_ between Holstein and Friesian or Jersey cows [18].

In the present study, we have demonstrated that polymorphisms in both, *SELP* and *SELE* were associated with differences in fertility in the sampled dairy cows. It must be noted that such associations do not infer causation as the SNP identified may be in LD with other genes, which may have also influence the traits concerned. We therefore checked the genes along BTA16 for 500 Kb around the selectin genes: these are *TRNAC-GCA*, *ATP1B1*, *NME7*, *BLZF1*, *CCDC181*, *SLC19A2*, *TRNAG-CCC*, *F5*, *[SELP*, *SELL*, *SELE]*, *METTL18*, *C16H1orf112*, *SCYL3*, *KIFAP3*. There is currently no available evidence to favor any of these as being more likely to influence fertility. We also investigated milk yield, as this has a negative genetic correlation with fertility in lactating animals [58, 59]. A major reason for studying selectin polymorphisms initially was the expectation that we might find associations with the incidence of mastitis. This was based on the proximity of the selectin gene cluster to a putative mastitis-related quantitative trait locus (QTL), the up-regulation of *SELL* and *SELP* in the mammary gland following experimental infection with either *S*. *uberis* or *E*. *coli* and the established role of selectins in granulocyte diapedesis during inflammation (reviewed by Chen et al. [60]). We initially analysed SCC using two approaches, calculating the mean SCC per lactation and using a mixed model in ASReml for all lactations and for the first lactation alone (data not shown) as well as the tertiary trait analysis described here which classified cows into three categories based on the criteria defined by Yoshida et al. [51]. SELP_Ex6_ was the only one of the eight SNP tested by each of these three methods which showed any significant association with SCC at P < 0.05, with animals carrying a G allele more likely to have a high SCC. These results indicated that the selectins are unlikely to be the causative genes for the putative mastitis QTL, at least in this population of Holstein Friesian cows. Although there were some significant associations between milk production and the polymorphisms analysed, these did not explain the fertility changes.

The SELP_Ex4-6_ haplotype block contains the missense SNP rs42312260 (SELP_Ex5_), which causes a Glu to Ala amino acid change in CR2. The three SNP located in this block were all in HWE. The SELP_Ex4_ TT homozygotes had shorter lives by around 4 months than for animals carrying at least one C allele and only 45% reached their third lactation, compared with around 60% for animals carrying a C allele. Poor fertility accounted for 63% of the culls. The AG heterozygotes for SELP_Ex6_ also experienced worse fertility than the GG homozygotes. The evidence therefore suggests that Glu/Ala substitution at rs42312260 in the SELP_Ex4-6_ haplotype block is associated with reduced conception rates in both heifers and cows. The effect became more pronounced in lactating animals, which experience worse fertility than heifers due to the competing nutrient requirements of milk production [4].

The longevity and fertility traits which we recorded are not identical to those used in other GWAS studies. We monitored each animal from birth until death, whereas the indices used by breeding organisations for longevity do not start until first calving, thus excluding any heifers which fail to conceive at all. The UK longevity trait is measured in lactations. As actual daughter survival data are not available for young bulls, information on type traits (feet, legs and udder), cell count and family are used to make the best possible predictions of lifespan [61]. For US Holsteins, the Productive Life (PL) is based on the total days of lactation up to 84 months of age, but excludes dry periods. Tsuruta et al. [62] compared three different PL evaluations with Herd Life (HL), which was defined as the total days from the first calving date to the last (culling) date (i.e. including dry periods). These different definitions of the phenotype will all impact on the results of earlier GWAS studies. In support of our findings, Cole et al. [63] reported a SNP rs110120157 (BAT16: 37505165) selected by GWAS, which was related with PL. The US Holstein cows in this study were genotyped using the BovineSNP50 Bead chip and the results analysed against 31 traits. This SNP was the closest to the selectin genes at a distance of about 500Kb, so it could potentially be in LD. Furthermore, this particular SNP was also significantly associated with several other highly relevant traits including somatic cell count, daughter pregnancy rate and daughter stillbirth rate. Another study [64] reported a QTL (BAT16:22.5–42.5Mb) related to calving ease, which covers the region occupied by the selectin genes. This was not a trait we recorded, but would be influenced by fetal size at birth, which in turn known is related to placental development in cattle [65,66].

The extensive evidence from the human, cited in the literature review above, suggests that SELL rather than SELP plays a key role in embryo attachment to the endometrium, although SELP is present during human placental development. In the cow SELP was up-regulated in the endometrium on days 20–22 after insemination and the ligand PSGL1 was present in the pre-implantation cow conceptus, coinciding with the time when attachment is normally initiated [42, 67]. It is therefore plausible that the effects reported associated with the Glu/Ala substitution in CR2 could be due to the mutation reducing the efficiency of placental attachment, so leading to early pregnancy losses. The repeating CRs in the SELP molecule are thought to be important in providing extra length, so helping to project the lectin domain above the cell membrane [68]. It is the N-terminal lectin domain, which contains the carbohydrate-binding site through which SELP interacts with PGSL1 and other ligands [69].

The pattern of results differed for the *SELP* SNP rs378218397 (SELP_Ex8_), which is a Val475Met variant locus in the CR4 and CR5 linking region. Bioinformatic analysis showed that this SNP was very close to a predicted N-acetyl-D-glucosamine glycosylation site [18]. SELP has two N-linked glycosylation sites at the interface of the CRs. Glycosylation may protect the exposed CR domain linking regions against proteolytic cleavage [70]. This SNP was located very close to the Val599Leu polymorphism in the human *SELP* gene, which is associated with soluble SELP concentrations, particularly in the African American population [29]. On the other hand, Subramanian et al. [71] provided evidence that the mutation Thr715Pro in human *SELP*, located in CR9, impairs terminal glycosylation of SELP in the Golgi, so reducing the amount of mature SELP produced. The human *SELP* gene has nine CR, compared with only six in cattle [18]. Both SELP_Ex8_ and SELP_Ex13_ tended to deviate from HWE, with cows carrying the A allele being under-represented. Theoretical calculations indicated that these differences in HWE would have become significant had we been able to genotype approximately 20 more cows, assuming the genotype proportions remained the same. For SELP_Ex8_ only four AA animals were found from 320 heifers genotyped at 6 months of age, of which only two calved. Subsequently the AG heterozygotes had worse fertility that the GG homozygotes. This strongly suggests that cows carrying the A allele had reduced fertility and that AA homozygous embryos are unlikely to survive prenatal development. This result does, however, need confirmation in another larger population and it also remains to be determined at what point in the pregnancy these predicted losses might occur.

In accordance with studies in humans [48], there were fewer polymorphisms in the bovine *SELL* and *SELE* genes compared to *SELP*. The three SNP investigated, forming another haplotype block, were all synonymous. Nevertheless, there is increasing evidence that synonymous mutations, which do not alter the amino acid sequence, are under selective pressure and can affect the function of translated protein through diverse cellular mechanisms. These include influencing splice sites, miRNA and exonic transcription factor binding sites, affecting mRNA stability and altering translational speed [72]. This second haplotype block showed some significant associations with both fertility and milk production, but not with SCC. The SNP rs110045112 in SELE_Ex14_ was not in HWE, with fewer GG homozygotes than expected. The GC heterozygotes took significantly longer than the CC homozygotes to conceive in the second lactation but also had higher 305d milk yields. *SELE* is expressed in the human decidua, cytotrophoblast and syncitiotrophoblast [36] but it has not to our knowledge been studied in the bovine placenta. This evidence suggests that changes in function of SELE are also associated with reduced embryo survival decreasing fertility although the regulatory mechanism remains to be elucidated.

Some of the fertility differences reported showed as differences in intervals to conception (the SELP_Ex4-6_ haplotype block), whereas others were mainly distinguished by a significant depletion in frequency of one homozygote (SELP_Ex8_ and SELP_Ex13_, SELE_Ex14_). Using an *in vitro* fertilization approach, some cattle genotypes have been associated with reduced fertilization and embryo survival rates during early pregnancy [73,74]. Charlier et al. [9] found 296 loss of function variants and 3,483 disruptive missense breed-specific variants in populations of Belgian beef and New Zealand dairy cattle. They identified nine embryo lethal mutations at frequencies of 1.2% to 6.6% in their populations, accounting for the losses of about 0.6% of all conceptuses. The data presented here suggest that selectin polymorphisms may also lead to embryos with lethal loss of function genotypes. The evidence that selectins also play an essential role in later placental development presents an additional dimension, as the differing genotypes between the maternal and fetal tissues involving both selectins and their ligands such as PGSL1 may also influence the likely success of a particular pregnancy. This area remains largely unexplored to date.

## Conclusions

This study has provided evidence that polymorphisms in *SELP* were associated with reduced fertility in the cow through likely changes in protein functionality, which may affect both attachment of the conceptus to the endometrium and embryo viability. A synonymous SNP in *SELE* was also associated with reduced fertility, although the mechanism has not yet been determined. Such associations do not provide definitive evidence of cause and effect but this information can nevertheless be used to improve breeding decisions in the Holstein cattle population. *SELP* is also highly polymorphic in women [48]. The SNP rs6127, located in CR7, has been associated with recurrent spontaneous abortion, with the risk increased by 2.65 and 4.96 respectively in the heterozygous and homozygous carriers [35]. Our results support the importance of normal SELP functionality in establishing and maintain a pregnancy, suggesting that genotyping of human couples who either fail to conceive or lose their pregnancies would be informative.

RVC Manuscript number PPH_01462.

## Supporting information

S1 Table Primers and restriction enzymes used for genotyping of bovine selectin SNP by PCR-RFLP. (DOCX)

S2 Table List of the individual SNP genotypes for the cows included in the study. (PDF)

S3 Table Fertility phenotypes for the cows included in the study. (PDF)

S4 Table Survival times for the cows included in the study. (PDF)

S5 Table Milk phenotypes for the cows included in the study. (PDF)
