# Supplementary material for: Polymorphisms in the selectin gene cluster are associated with fertility and survival time in a population of Holstein Friesian cows
Source: PLoS One. 2017 Apr 18;12(4):e0175555. doi: 10.1371/journal.pone.0175555 (PMC5395145; doi:10.1371/journal.pone.0175555)
Supplement: S1 Table — (DOCX) [file pone.0175555.s001.docx]

**S1Table.** Primers and restriction enzymes used for genotyping of bovine selectin SNP by PCR-RFLP.

| SNP | Primer(5’-3’) | Ann. temp (℃) | Enzyme | Alleles | PCR-RFLP fragment length (bp) |
| --- | --- | --- | --- | --- | --- |
| SELP_Ex4_  rs110033243 | For: GCTGGAAATAAACACCAGTGAAAGT  Rev: GCATGAGGATCAAGAATTAAACTCC | 57 | Mwol | C/T | TT: 794  TC: 794/517/277  CC: 517/277 |
| SELP_Ex5_  rs42312260 | For: CTGCTCCAGTATTCTTGCC  Rev: GGTTTCATCTGTGCTCCC | 64 | BstBI | C/A | CC: 449  CA: 145/304/449  AA: 145/304 |
| SELP_Ex6_  rs137027551 | For: GATACTTTTCTCATCCTCCTTGTTA  Rev: ATCCCGTCGCTTGTGGT | 57 | Hpy188I | A/G | AA: 40/377  AG 40/120/377/497  GG: 40/120/377 |
| SELP_Ex8_  rs378218397 | For: GTGGATGGTTTAGTTGCTAGGTT  Rev: TGGGATTGCTGTGGGTTT | 57.6 | NdeI | G/A | GG: 725bp  GA: 725/464/261  AA: 464/261 |
| SELP_Ex13_  rs211179622 | For: GTGACCTCGTCCAATCCC  Rev: CCTCCTGCCTTCCAACAA | 57 | PstI | T/A | AA: 526  AG: 200/326/526  GG 200/326 |
| SELL_Ex3_  rs109966956 | For: TGTCAGCTTCCTCGTCTC  Rev: GTTCGTTCCCAGCACTCT | 57 | BsmI | G/A | GG:547  GA:182/365/547  AA:182/365 |
| SELL_Ex4_  rs41803 917 | For: CCCCTAAATGTTATAGAGTATCAG Rev: TATTCCAATGCCAATGCTCT | 57.6 | AvaI | T/C | TT:589 CT:245/344/589 CC:245/344 |
| SELE_Ex14_  rs110045112 | For: GACCGTGCTGGGAGATAA Rev: AACTCCTCGGACCACAGA | 57 | Tth111I | C/G | CC:572 CG:212/360/572  GG: 212/360 |
