# Supplementary material for: Polymorphisms in the selectin gene cluster are associated with fertility and survival time in a population of Holstein Friesian cows
Source: PLoS One. 2017 Apr 18;12(4):e0175555. doi: 10.1371/journal.pone.0175555 (PMC5395145; doi:10.1371/journal.pone.0175555)
Supplement: S2 Table — (PDF) [file pone.0175555.s002.pdf]

S2 Table. List of the individual SNP genotypes for the cows included in the study.

Cow ID	Farm ID	SELP-EXON13 rs211179622	SELP-EXON8 rs378218397	SELP-EXON6 rs137027551	SELP-EXON5 rs42312260	SELP-EXON4 rs110033243	SELL_EXON4 rs41803917	SELL-EXON3 rs109966956	SELE-EXON14 rs110045112
607	11	T T	G G	A G	A C	C T	C C	A G	C C
845	17	A T	G G	G G	C C	C T	C T	G G	C G
847	17	A T	G G		C C	C T		A G	C C
848	17	A T	G G	G G	C C	C C	C T	G G	C G
849	3	T T	G G	G G	C C	C C	C C	A A	C C
850	17	T T	A G	G G	A A	T T	C C	A G	C C
851	3	T T	G G	G G	A C	C T	C C	G G	C C
852	20	A A	G G	G G	C C	C C	T T	G G	C G
1342	5	T T	G G	A G	A A	T T	C C	A A	C C
1343	3	T T	G G	G G	A C	C T	C C	G G	C C
1344	3	A T	A G	A G	A C	C T	C T	A G	C G
1345	18	T T	A G	A G	A A	T T	C C	A A	C C
1347	21	T T	A G	A A	A A	T T	C C	A A	C C
1348	11	T T	A G	G G	A C	C T	C T	G G	C G
1349	3	T T	A G	A G	A A	T T	C C	A A	C C
1350	21	T T	A G		A A	T T	C C	A A	
1352	18	T T	G G	G G	C C	C C	C T	A G	C G
1353	5	A T	G G	G G	C C	C C	C T	A G	C G
1354	6	T T	G G	A A	A A	T T	C C	A A	C C
1355	3	T T	A G	G G	A A	T T	C C	A G	C C
1356	6	A T	G G	A G	A C	C T		A G	C G
1357	21	T T	G G	G G	A C	C T	C C	A G	C C
1360	6	A T	G G	G G	C C	C C	C T	A G	C G
1361	21	T T	G G	A G	A C	C T	C C	A A	C C

1362	3	T T	A G	A G	A C	C T	C C	A A	C C
1363	3	T T	A G	A G	A C	C T	C C	A A	C C
1699	15	A T	G G	G G	C C	C C	C T	A G	C G
1701	15	A T	G G		C C	C C	C T	A G	
1702	2	T T	G G	G G	C C	C C	C T	A G	C G
1703	2	T T	A G	A G	A C	C T	C C	A A	C C
1704	2	T T	G G	A G	A C	C T	C C	A A	C C
1706	10	T T	G G	G G	C C	C C	C T	G G	C G
1709	4								
1710	6	A A	G G	G G	C C	C C	T T	G G	G G
1712	4	A T	G G	G G	A C	C T	C T	G G	C G
1714	6	A T	G G	G G	C C	C C	C T	G G	C G
1715	6	A T	G G	G G	C C	C C	C T	A G	C G
1717	6	A T	A G	A G	A C	C T	C T	A G	C G
1720	16	A T	G G	G G	C C	C C	C T	A G	C G
1723	21	A T	A G	G G	A C	C T	C T	G G	C G
1725	16	A T	G G	G G	C C	C C	C T	A G	C G
1726	16	A T	G G		C C	C C	C T	A G	
1727	14	T T	G G		A A	T T	C C	A A	
1729	9	T T	G G		A C	C T	C C	A A	C C
1731	1	A T	A G	G G	A C	C T	C T	A G	C G
1732	11	T T	G G	A G	A C	C T	C C	A G	C C
1733	1	A T	G G	G G	C C	C C		G G	C G
1734	1	T T	G G		C C	C C	C C	A A	
1735	11	T T	A G		A A	T T	C C	G G	
1736	13	A T	A G	G G	A C	C T	C T	A G	
1738	19	T T	A G		A C	C T	C C	A A	C C
1739	19	T T	A G	A G	A C	C T	C C	A G	
1740	18	T T	G G	A G	A C	C T	C C	A G	C C
1744	20	A T	G G		A C	C C	C T	G G	C G

1745	20	T T	G G	A G	A C	C T	C T	A G	C G
1746	15	A T	A G	G G	C C	C C	C T	A G	
1748	15	T T	G G	G G	A C	C T	C C	A G	C C
1750	2	A T	A G	A G	A C	C T	C T	A G	C G
1751	2	T T		A A	A A	T T	C C	A A	C C
1753	6	A T	G G	G G	C C	C C	C T	A G	C G
1755	5	T T	G G	G G	C C	C C	C T	A G	C G
1757	4	A T	A G	G G	A C	C T	C C	A A	C C
1758	4	T T	A G	A G	A A	T T	C C	G G	C C
1760	4	T T	G G	G G	A C	C T	C C	A G	C C
1761	5	A T	G G	A G	A A	T T		A A	C C
1762	6	T T	G G	G G	A C	C T	C C	G G	C C
1763	6	T T	G G	A G	A A	T T	C T	G G	C G
1764	6	T T	G G	G G	A C	C T	C C	A G	C C
1765	5	T T	G G	A G	A A	T T	C C	A A	C C
1766	6	T T	A G	A A	A A	T T	C C	A A	C C
1767	17	T T	G G	G G	C C	C C	C T	A G	C G
1768	17	T T	G G		C C	C C	C T	A G	
1770	16	T T	G G	A G	A C	C T	C C	A G	C G
1772	21	T T	A G	G G	A C	C T	C C	A G	C C
1773	21	A T	G G	A G	A C	C T	C T	A G	C G
1776	14	T T	G G	A A	A A	T T	C C	A A	C C
1777	14	T T	G G	A A	A A	T T	C C	A A	C C
1781	11	A T	G G	G G		C T	C T	G G	C G
1782	3	A T	A G	A G	A C	C T	C T	A G	C G
1783	1	A T	G G		C C	C C	C T	G G	
1785	11	T T	G G	G G	C C	C C	C C	A G	C C
1786	3	A T	G G	A G	A C	C T	C T	A G	C G
1787	12	A T	A G	A G	A C	C T	C C	A G	C C
1789	19	T T	A G	A G	A C	C T	C C	A A	C C

1790	19	A T	A G	G G	A A	T T	C C	A G	C C
1791	19	T T	A G		A C	C T	C T	A G	
1792	19	T T	G G		A A	T T			
1796	15	T T	A G		A A	T T	C C	A A	
1798	15	T T	A G		A A	T T	C C	A A	
1799	2	T T	A G		A C	C T	C C	A G	
1800	2	A T		A G	A A	T T	C C	A A	C C
1801	2	A T	A G	A G	A C	C T	C T	A G	C G
1802	10	T T	A G	A G	A C	C T	C C	A A	C C
1804	5	T T	A G	A G	A C	C T	C C	A A	C C
1810	4	T T	G G	G G	C C	C C	C C	A G	C C
1811	5	A T	A G	A G	A A	T T	C C	A G	C C
1812	4	A T	G G	A G	A C	C T	C T	G G	C G
1813	5	A A	G G		A C	C T	T T	G G	C C
1816	16	T T	G G	G G	C C	C C	C C	A A	C C
1817	16	T T	G G	A G	A C	C T	C C	A A	C C
1819	14	T T	A G	A G	A C	C T	C C	A A	C C
1820	14	A T	G G	A G	A A	T T	C C	A G	C C
1821	14	T T	A G	A G	A C	C T	C C	A A	C C
1822	14	T T	A G	A G	A C	C T	C C	A A	C C
1824	11	T T	A G	A G	A C	C T	C C	A G	C C
1825	1	T T	G G	G G	A C	C T	C T	G G	C G
1826	1	T T	A G	G G	A C	C T	C C	G G	C C
1827	3	A T	G G	A G	C C	C C	C C	G G	C C
1828	13	A T	G G	G G	C C	C C	C T	G G	C G
1829	12	T T		A A	A A	T T	C C	A A	C C
1830	19				A A	T T	C T	A G	C G
1831	19	T T		A G	A C	C T	C T	A A	C G
1832	19	A T	G G		A C	C T	C T	A G	C G
1834	18	A T	G G	A G	A C	C T		A A	C C

1835	15	T T	G G	G G	C C	C C	C C	A A	C C
1837	15	T T	A G		A A	T T	C C	A A	
1838	2	T T	A G	A A	A A	T T	C C	A A	C C
1839	10	T T	A G	A G	A C	C T	C C	A A	C C
1840	5	A T	G G	G G	C C	C C	T T	G G	C G
1841	10	T T	G G		C C	C C	C C	A A	
1843	6	T T	G G	G G	A C	C T	C C	A G	C C
1845	5	A A	G G	G G	C C	C C	T T	G G	G G
1846	6	T T	G G	G G	C C	C C	C C	A A	C C
1847	4	A T	G G	G G	C C	C C	C T	G G	C G
1848	5	A T	G G	G G	C C	C C	C T	A G	C G
1849	4	A T	G G		C C	C C	C C	A G	
1850	4	T T	G G	A G	A A	T T	C C	A G	C C
1852	16	A T	G G	G G	C C	C C	C T	A G	C G
1853	21	T T	A G		A A	T T	C C	A G	
1854	21	A T	G G		C C	C C	C T	A G	
1855	14	A T	A G	A G	A C	C T	C T	A G	C G
1857	14	A T	G G	A G	A C	C T	C C	A A	C C
1861	3	A T	G G	A G	A C	C T	C T	A G	C G
1862	3	A T	A A	G G	A C	C T	C T	A G	C G
1863	3	T T	G G	A G	A A	T T	C C	A G	C C
1864	13	A T	G G	G G	C C	C C	T T	G G	
1865	13	T T	G G	G G	C C	C C	C C	G G	C C
1866	3	A T	A G		C C	C C		A G	C C
1867	13	T T	G G	A G	A C	C T	C C	A A	C C
1868	13	A T	G G	G G	A C	C T	C T	G G	C G
1869	12	T T	A G	A G	A C	C T	C T	A G	C G
1870	19	A T	A A	A G	A A	T T	C C	A A	C C
1871	19	A T	G G		C C	C C	C T	G G	C G
1874	18	A A	G G	G G	C C	C C	T T	G G	G G

1875	18	T T	G G	G G	C C	C C	C C	A A	C C
1879	2	T T	G G		C C	C C	C C	A G	C C
1880	2	T T	A G	A A	A A	T T	C C	A G	C C
1881	2	T T	G G		A A	T T	C C	A G	
1883	10	A T	G G		A C	C T	C T	G G	
1884	6	T T	G G	A A	A A	T T	C C	A A	C C
1885	4	A T	G G	G G	C C	C C	C T	G G	C G
1889	6	A T	A G	A G	A C	C T	C T	A G	C G
1890	4	A T	G G	G G	C C	C C	C C	A A	C C
1891	4	T T	G G	A G	A C	C T	C C	A G	C C
1893	4	A T	G G	G G	C C	C C	C T	A G	C G
1896	17	T T	G G	G G	C C	C C	C C	A A	C C
1898	21	A T	G G	G G	C C	C C	C T	A G	C G
1899	14	T T	G G	G G	C C	C C	C C	A A	C C
1900	14	T T	G G	A A	A A	T T	C C	A A	C C
1902	14	T T	A G	A A	A A	T T	C C	A A	C C
1903	14	T T	A G	A A	A A	T T	C C	A A	C C
1904	14	T T	G G	G G	C C	C C	C C	A G	C C
1907	1	T T	A G	A G	A A	T T	C C	A A	C C
1908	11	A T	G G	G G	A C	C T	C T	G G	C G
1909	11	A T	G G	G G	C C	C C	C T	G G	C G
1910	3	T T	G G	A G	A C	C T	C C	A G	C C
1911	13	T T	G G		C C	C C	C C	A G	C C
1912	12	T T	G G	G G	C C	C C	C C	A G	C C
1913	12	A T	A G	A A	A A	T T	C C	A A	C C
1914	12	T T	G G	G G	C C	C C	C C	A A	C C
1915	12	T T	A G		A C	C T	C C	A G	
1917	19	T T	G G		A C	C T	C C	A G	C G
1918	19	A T	G G	G G	C C	C C	C C	A G	C C
1919	18	T T	G G		A C	C T	C C	G G	

1922	20	A T	G G	G G	C C	C C	C T	A G	C G
1925	15	T T	G G	A G	A C	C T	C C	A G	C C
1928	2	T T		A A	A A	T T	C C	A G	C C
1929	2	A T	A G	A G	A C	C T	C T	A G	C G
1931	10	A T	A G		C C	C T	C C	A A	C C
1932	10	T T	A G		A C	C T	C C	A A	C C
1933	6	T T	G G	G G	A C	C T	C C	A G	C C
1935	5	A T	G G	A G	A C	C T	C T	A G	C G
1937	5	T T	G G	G G	A C	C T	C T	G G	C G
1938	6	T T	G G	A G	A C	C T	C C	A A	C C
1939	5	T T	G G	G G	C C	C C	C C	A G	C C
1940	6	A T	G G	G G	C C	C C	T T	A G	C G
1941	4	T T	G G	G G	C C	C C	C T	A G	C G
1942	4	A T	G G	G G	C C	C C	T T	G G	C G
1943	5	T T	G G		C C	C C	C T	A A	
1945	17	A A	G G	G G	C C	C C	C C	A A	C C
1946	17	T T	G G	G G	C C	C C	C C	A A	C C
1947	21	A T	G G		C C	C C	C T	G G	
1949	14	T T	G G		C C	C C		A G	
1952	1	T T	A G	A G	A C	C T	C C	A A	C C
1953	1	T T	G G	A G	A A	T T	C C	A G	C C
1954	3	T T	G G	G G	C C	C C	C C	A A	C C
1955	13	T T	G G		A C	C T			C C
1956	12	T T		G G	A C	C T	C C	G G	C C
1957	12	T T	G G	A G	A C	C T	C C	A G	C C
1958	1	T T	G G	G G	A C	C T	C T	A G	C G
1962	19	A T			A C	C T			C C
1963	19	T T	G G		A A	T T	C C	A A	C C
1966	15	T T	G G	G G	A C	C T	C C		C C
1967	15	T T	G G	A G	A C	C T	C C	A A	C C

1969	4	A T	G G		A C	C T	C T	G G	
1973	5	T T	G G	A G	A C	C T	C C	A A	
1974	4	T T	G G		C C	C C	C C	A A	
1975	4	A T	G G	A G	A C	C T	C T	A G	C G
1977	5	T T	G G	G G	C C	C C	C C	A G	C C
1978	6	A T	A G	A G	A A	T T	C C	A A	C C
1979	4	T T	G G	A G	A C	C T	C C	A A	C C
1980	17	T T	A A	A G	A A	T T	C C	A G	C C
1983	21	T T	A G	G G	A A	T T	C C	A G	C C
1985	14	T T	A G	A G	A C	C T	C T	A G	C G
1986	21	T T	A G		A A	T T		A G	
1987	14	T T	A G	A A	A A	T T	C C	A A	C C
1991	11	A T	G G	A G	A C	C T	C T	A G	C G
1992	11	A T	G G	G G	C C	C C	C T	G G	C G
1993	1	T T	A G		A C	C T	C T	A G	C G
1994	11	T T	G G	A G	A C	C T	C C		C C
1995	3	T T	A G	G G	C C	C C	C T	A G	C G
1996	1	T T	G G		A A	T T	C C	A G	
1998	11	T T	G G	G G	A A	T T	C C	G G	C C
1999	12	A T	G G		C C	C C	C T	G G	
2000	12	A T	G G	A A	A A	T T	C C	A A	C C
2001	1	T T	G G	G G	A C	C T	C T	A G	
2003	19	T T	G G	G G	C C	C C	T T	G G	G G
2004	19				A A	T T	C C	A G	C C
2005	19		G G		C C	C C	T T	A G	C G
2083	2	T T	G G	A G	A C	C T	C C	A A	
2087	16	A T	G G	G G	C C	C C	C T	G G	C G
2088	16	A T	A G	G G	A A	T T	C T	A G	C G
2089	21	A T	G G	G G	A C	C T	C C	A G	C G
2093	11	T T	A G	A G	A A	T T	C C	A G	C C

2095	1	A T	A G	A G	A C	C T	C C	A G	C G
2096	3	T T	G G	A G	A C	C T	C C	G G	C C
2097	13	A T	G G	G G	C C	C C	C T	A G	C G
2102	19	A T		A G	A C	C T	C T	A G	C G
2103	19	T T	A G		A C	C T	C C	A G	
2104	18	T T	A A	A G	A A	T T	C C	A A	C C
2107	17	A T	G G	G G	A C	C T	T T	G G	G G
2109	16	A T	A G		A C	C T	C T	A G	C G
2110	14	A T	G G		C C	C C	C T	A G	
2111	16	A T	G G	A G	A C	C T	C T	A G	C C
2113	14	A T	G G	G G	A C	C T	C T	G G	C G
2115	1	A T	A G	A G	A C	C T	C T	A G	C G
2116	1	A T	A G	A G	A C	C T	C T	A G	C G
2117	1	A T	A G		A A	T T	C C	A A	C C
2118	13	A T	A G	G G	A C	C T	C T	A G	C G
2119	13	T T		G G	A C	C T	C C	A A	
2120	12	T T		A G	A C	C T	C C	A G	C C
2121	12	A T	A G	A G	A C	C T	C C	A G	C C
2122	8	T T	A G	G G	A C	C T	C C	A A	C C
2125	18				A C	C T	C C	G G	C C
2126	18	T T	G G	A G	A C	C T	C C	G G	C C
2128	16	T T	A G	A G	A A	T T	C C	A A	C C
2130	16	A T	A G	G G	A C	C T	C T	A G	C C
2131	21	A T	G G	G G	C C	C C	T T	G G	G G
2132	16	A A	G G	G G	C C	C C	T T	G G	G G
2133	11	A T	A G	A G	A C	C T	C T	A G	C G
2134	11	A A	G G	A G	C C	C C	C T	A G	C G
2135	1	T T	G G	G G	A A	T T	C T	G G	C C
2136	3	A T	G G	A G	A C	C T	C T	A G	C G
2137	1	A T	G G		A C	C T		G G	C C

2138	13		G G		C C	C C	T T	G G	C G
2139	13	A T	A G	A G	A C	C T	C T	A G	C G
2140	13	T T	G G	A G	A C	C T	C C	A A	C C
2141	12	A T		A G	A C	C T	C C	A G	C C
2148	18	T T	G G	A G	A C	C T	C C	G G	C C
2150	4	T T	G G		A C	C T		A G	
2152	16	T T	A G		A A	T T	C C	A A	
2153	16	A T	A G	A G	A C	C T	C T	A G	C G
2154	21	A T	G G	G G	C C	C C	C T	A G	C G
2155	14	T T	G G	A G	A C	C T	C C	A A	C C
2156	14	T T	G G		A C	C T		G G	C G
2159	11	A T	G G	G G	C C	C C	C C	A G	C C
2161	3	A T	G G		C C	C C	T T	G G	
2162	1	T T	G G	A G	A C	C T	C C	A G	C C
2163	1	A T	G G		A C	C T	C T	G G	
2164	1	T T	G G	A G	A C	C T	C C	A G	C C
2165	3	T T	A G	A G	A C	C T	C T	A G	C G
2166	13	T T	G G		C C	C C		G G	
2167	13	T T	A G	A G	A A	T T	C C	A A	
2168	12	T T	G G	A G	A C	C T	C C	A A	C C
2169	1	A T	A G	G G	A C	C T	C T	A G	C G
2173	19	T T	G G	A G	A A	T T	C T	A G	C G
2174	18	T T	G G	A G	A C	C T	C C	G G	C C
2175	18	T T	G G	G G	C C	C C	C C	G G	C C
2177	4	T T	G G		C C	C C	C C	A A	
2178	21	T T	G G	A A	A A	T T	C C	A G	C C
2179	16	A A	G G	G G	C C	C C	T T	G G	C G
2180	16	A A	G G	A G	A C	C T	C T	A G	C G
2184	1	A T	A G	G G	A C	C T	C T	A G	C G
2185	1	A T	G G	A G	A C	C T	C T	A G	C G

2186	13	A T	G G	A G	A C	C T	C T	A G	C G
2187	13	A T	A G	A G	A C	C T	C T	A G	C G
2188	13	A T	G G	G G	C C	C C	C T	A G	C C
2189	13	T T	A G	A A	A A	T T	C C	A A	C C
2190	1	T T	G G	A A	A A	T T	C C	A A	C C
2191	12	A T	A G	A G	A C	C T	C T	A G	C G
2194	8	A T	G G	A G	A C	C T	C C		C G
2195	19	T T			A C	C T	C T	A G	C G
2196	18	A T	G G	A G	A C	C T	C T	G G	C G
2198	20	A A	G G		C C	C C	T T	G G	
2200	14	A T	G G	G G	A C	C T	C C	A A	
2201	21	A T	G G	G G	C C	C C	C T	G G	C G
2202	16	T T	A G	G G	A C	C T	C C	A G	C C
2205	11	T T	A G	A A	A A	T T	C C	A A	C C
2206	1	T T	A G		A A	T T	C C	A G	C C
2207	1	A T	A G	G G	A C	C T	C T	A G	C G
2209	3	A T	A G	G G	A C	C T	C T	A G	C G
2210	13	T T	A G	G G	A C	C T	C T	A G	
2211	13	T T	G G	G G	A C	C T	C C	G G	C G
2212	13	A T	G G	G G	C C	C C		G G	
2213	13	T T	G G	A G	A C	C T	C C	A A	
2214	12	T T	G G	A A	A A	T T	C C	A A	C C
2215	12	T T	G G	G G	A C	C T	C C	A G	C C
2219	19	T T	G G	A G	A C	C T	C T	A G	C G
2220	18	T T	G G		A C	C T	C T	G G	
2221	18	T T	A G	A A	A A	T T	C C	A A	C C
2222	18	T T	G G	A G	A C	C T	C C	A G	C C
2224	4	T T	G G	G G	C C	C C	C C	A A	C C
2225	5	T T	G G	A G	A C	C T	C C	A A	C C
2227	14	T T	G G	A G	A C	C T	C C	A A	C G

2228	14	T T	G G	A A	A A	T T	C C	A A	C C
2229	14	A T	G G		C C	C C	C T	A G	C G
2232	11	A T	A G	G G	C C	C C		A G	C C
2233	1	A T	A G	A G	A C	C T	C T	A G	C G
2234	3	T T	G G	G G	C C	C C	C C	G G	C C
2235	13	T T	G G	A G	A A	T T	C C	A G	C C
2236	13	A T	A G	A G	A A	T T	C C	A A	C C
2237	12	A T		A G	A C	C T	C C	A G	C C
2239	8	T T	A G	A G	C C	C T		A G	C G
2241	18	T T	G G	G G	C C	C C	C C	A G	C C
2242	18	A T	G G	A G	A C	C T		G G	C G
2250	13	A T	G G	G G	A C	C T	C T	G G	C G
2251	20	T T	G G		A A	T T	C C	A G	
