# Supplementary material for: Polymorphisms in the selectin gene cluster are associated with fertility and survival time in a population of Holstein Friesian cows
Source: PLoS One. 2017 Apr 18;12(4):e0175555. doi: 10.1371/journal.pone.0175555 (PMC5395145; doi:10.1371/journal.pone.0175555)
Supplement: S3 Table — (PDF) [file pone.0175555.s003.pdf]

S3 Table. Fertility phenotypes for the cows included in the study#.

Cow ID	Farm ID	AFC	DC_L1	CI_L1-2	ASC	DC_L2	CI_L2-3	No. calvings
607	11	6.7429	4.4773	5.9135	7.1050	4.1431	5.8289	3
845	17	6.5323	4.7274	5.9764	6.9856	4.5326	5.9216	3
847	17	6.4998	4.9836	6.0474	6.9921	4.0431	5.8141	3
848	17	6.5177	4.1109	5.8260	6.9236	4.4998	5.9135	3
849	3							0
850	17	6.5582	4.3175	5.8493	6.9584	4.2195	5.8665	3
851	3	6.9801	4.7958	6.0186	7.3038	4.1897	5.8348	4
852	20	6.4615	5.1299	6.1003	6.9903	4.9127	5.9738	3
1342	5							0
1343	3							0
1344	3	7.0211	4.7449	5.9375	7.3126	5.0752	6.1092	3
1345	18	6.6619						1
1347	21	7.0103	4.2047	5.8522	7.2834	4.2905	5.8636	3
1348	11	6.6758						1
1349	3							0
1350	21							0
1352	18	6.5903	4.4543	5.9054	6.9985	5.2523	6.1759	4
1353	5	6.6733	3.9890					1
1354	6	6.5043	4.9127	6.0544	6.9976	4.5747	6.1048	4
1355	3	7.0076	5.0876	6.1048	7.3479	4.5539	5.9269	3
1356	6	6.6554	4.1271	5.8081	7.0121	4.6347	5.9428	3
1357	21							0
1360	6	6.6490	4.0431	5.7869	7.0012	5.3566	6.2086	4
1361	21	7.0238	3.9890	5.8348	7.2896	4.1271	5.8406	4
1362	3	7.0076	4.4886	5.9269	7.2998	4.7362		2
1363	3	7.0076	4.3041	5.8972	7.2923	4.0604	5.8319	3
1699	15	6.7346	4.4308	5.9026	7.0959	5.0999		2
1701	15	7.0834	5.4161	6.2206	7.4354	4.2341	5.8551	3
1702	2	6.7604	4.2485	5.8522	7.0992			2
1703	2	6.6908	5.3033	6.3784	7.2399	4.6347	5.9480	3
1704	2	6.6834						1
1706	10	6.5834	6.0890	6.5862	7.2779	5.0938	6.0936	4
1709	4	6.5876	4.2905	5.8608	6.9819			2
1710	6							0
1712	4	6.5667	4.3438	5.4553	6.8512			2
1714	6	6.5862	4.1897	5.8406	6.9745	4.1589		2
1715	6	6.5439	4.3041	5.8833	6.9603	4.5218	5.9108	4
1717	6	6.5667	4.5539	5.9108	6.9847	4.0604	5.6937	3

1720	16	6.7069	4.0604	5.8171	7.0510	5.3132	6.1862	4
1723	21	6.5985						1
1725	16	6.8255	4.1431	5.8319	7.1405			2
1726	16	6.8287	4.5951	5.9584	7.1785			2
1727	14	6.5667	4.0775	5.8260	6.9565	4.4308	5.9081	3
1729	9	6.5793						1
1731	1	6.7441	4.8283	5.9506	7.1172	3.8918	5.8081	4
1732	11	6.7673	4.5109	5.9243	7.1253			2
1733	1	6.7166	4.1109	5.8319	7.0622	4.8283	6.0039	3
1734	1	6.8405	4.9767					1
1735	11	6.8156	4.1897	5.8579	7.1405	4.5539	5.9349	3
1736	13	6.8341	4.6052	5.9349	7.1755	5.4027	6.2186	3
1738	19	6.7569	5.2417	6.0868	7.1701	4.5850	5.8889	3
1739	19	6.6320	4.6052	5.9189	7.0309	4.6728		2
1740	18	6.6346	4.0604	5.8230	7.0022	4.1431	5.8493	4
1744	20	6.4998	5.2257	6.1442	7.0309			2
1745	20	6.5073	4.5951	5.9402	6.9565	4.2905	5.8493	3
1746	15	6.7719	3.9890	5.7777	7.0867	4.7449	5.9610	5
1748	15	6.6859	4.8598	6.0234	7.1017	4.5951	5.9428	4
1750	2	6.6771	4.6444	5.9480	7.0707			2
1751	2	6.7286	5.1874	6.1291	7.1663	4.8122	6.0039	5
1753	6	6.5482	4.6444	5.9661	6.9921	4.8903	6.0307	4
1755	5	6.7358						1
1757	4							0
1758	4							0
1760	4	6.5250	6.0403	6.5554	7.2335	4.3307	6.2265	3
1761	5	6.5568	5.5134	6.2653	7.1148	4.2627		2
1762	6	6.6134	4.7791	5.9789	7.0388	4.1897	5.8493	3
1763	6	6.4892	6.0890	6.5765	7.2269	5.0039	5.9738	3
1764	6	6.5425	4.3567	5.8972	6.9641	5.4027	6.2146	3
1765	5	6.6254	4.2905	5.8721	7.0112	4.9416	6.0521	5
1766	6	6.6147	5.9189	6.4831	7.2442	4.7274	5.9814	4
1767	17	6.6821	4.1589	5.8289	7.0370			2
1768	17	6.6720	4.8828	6.0210	7.0917		5.4250	3
1770	16	6.7776						1
1772	21	6.6359	4.1431	5.8721	7.0184	4.1109	5.8522	4
1773	21	6.5751	4.4886	5.9189	6.9930	3.7842	5.7900	4
1776	14	6.5779	4.9698	6.0981	7.0596	4.9200	6.0426	4
1777	14	6.5779	4.8675	6.0210	7.0309	5.3279	6.1903	3
1781	11	6.7662	4.7362					1
1782	3	7.0031	4.6913					1

1783	1	6.7202						1
1785	11	6.7334	3.7377	5.7494	7.0510	5.5294	6.2804	3
1786	3							0
1787	12	6.8330	5.6699	6.3544	7.3152	4.6250	5.9480	3
1789	19	6.6399						1
1790	19	6.6026	4.3175	5.8636	6.9930	5.2575	6.1269	4
1791	19	6.6970	4.7274	5.9713	7.0917			2
1792	19	6.6908	4.7536	5.8944	7.0630			2
1796	15	6.8002						1
1798	15	6.6821	4.3438	5.8636	7.0475	5.6058	6.3297	3
1799	2	6.6490	4.7185	6.2577	7.1655	4.3438	6.2066	3
1800	2	6.7452	4.3944	5.8944	7.1009			2
1801	2	7.1357	4.5539	5.9322	7.3982	5.1761	6.1356	3
1802	10	6.6134	4.8203	6.0307	7.0570	4.1109	5.8201	5
1804	5	6.6012	4.7791	6.4846	7.2378			2
1810	4	6.5352	4.6347	5.9454	6.9763	4.6913	5.9216	3
1811	5	6.6529	4.7362	5.9789	7.0648	4.7362	5.9402	4
1812	4	6.6267	4.1897	5.8493	7.0049	4.7958	5.9940	4
1813	5	6.6093	4.8978	6.0162	7.0493	4.4886	5.8805	5
1816	16	6.7105						1
1817	16	6.6644	4.1589	5.8493	7.0309	3.7842	5.7900	4
1819	14	6.6093	4.2485	6.1985	7.1180	4.3820	5.8522	3
1820	14	6.8544	5.1818	6.0868	7.2356			2
1821	14	6.5971	4.5539	5.9135	7.0058	4.0431	5.7930	4
1822	14	6.5468						1
1824	11	6.8320	4.8520	6.0259	7.2012	4.3820		2
1825	1	6.7117	4.2047	5.8551	7.0656	4.1109	5.8348	5
1826	1	6.7032	5.1417	6.1247	7.1483	5.8141	6.4362	3
1827	3	6.9810	4.7362	6.0234	7.3059	4.4427	6.1696	3
1828	13	6.5624	4.9345	6.0307	7.0246	5.1358	6.1181	3
1829	12	6.8309						1
1830	19	6.5820						1
1831	19	6.5554	4.1744	5.8201	6.9470			2
1832	19	6.5596	4.0254	5.7961	6.9422	4.9836	6.0497	5
1834	18	6.5497						1
1835	15	6.7558	4.2905	5.8551	7.0967	4.3944	5.8749	5
1837	15	6.6503	5.6937	6.3596	7.2086	3.8286	5.8111	3
1838	2	6.7464	4.7449	5.9610	7.1221	3.9890	5.8377	5
1839	10	6.9985	5.0434	6.0684	7.3311		6.4846	3
1840	5	6.5624	4.9767	5.9940	7.0112	4.6728	5.9506	3
1841	10	6.6093	4.9273	6.0426	7.0588	4.3438	5.8665	5

1843	6	6.5985	4.2485	5.8579	6.9884	5.3471	6.1841	5
1845	5							0
1846	6	6.5073	4.9200	6.0259	6.9884			2
1847	4	6.5132	4.2195	5.8464	6.9276	5.2523	6.1291	4
1848	5	6.5723	4.6913	5.9940	7.0175	5.1985	6.1181	4
1849	4	6.7069	4.3307					1
1850	4	6.5695	4.0431	5.8289	6.9594	4.6728	5.9454	3
1852	16	6.7968	4.6634	5.9428	7.1515			2
1853	21	7.0238	4.6540	5.9428	7.3159			2
1854	21							0
1855	14	6.6080	5.9216	6.4892	7.2435	4.8040	6.0113	4
1857	14	6.6067	3.9890	5.7991	6.9754	3.9703	5.7900	5
1861	3							0
1862	3	6.9875	4.7791	6.0088	7.3065	4.3567	5.8999	3
1863	3	6.9921	4.3041	5.8777	7.2759	4.3307	5.8916	4
1864	13	6.6695	5.0938	6.0936	7.1156	4.2341	5.8551	4
1865	13	6.6970	5.2575	6.1420	7.1507			2
1866	3	7.0022	5.0752	6.1048	7.3441	4.4308	5.9108	3
1867	13	6.8112	4.8203	5.9940	7.1770	4.0254	5.7652	3
1868	13	6.6067	5.0239	6.0544	7.0613	5.9480	6.5043	3
1869	12	6.7719						1
1870	19	6.7696						1
1871	19	6.5765	4.9836	6.0497	7.0405	5.0499	6.3561	3
1874	18	6.6682	4.3820					1
1875	18	6.6720	4.8122	6.0064	7.0867	3.8918	5.7746	5
1879	2	6.7639	4.8978	6.0162	7.1515	4.7095	5.8777	5
1880	2	6.7776	4.8040	5.9940	7.1538	4.8122	6.1738	3
1881	2	6.6053	5.1358					1
1883	10	6.9939	4.9273	5.6204	7.2196	5.2204	6.1115	4
1884	6	6.5221	4.7362	5.9584	6.9726			2
1885	4	6.5958						1
1889	6	6.6053	4.9488	6.0450	7.0570	4.1431	5.8493	5
1890	4	6.5862	4.9972	6.0661	7.0527	5.6095	6.3172	3
1891	4	6.5596	4.2195	5.8665	6.9651			2
1893	4	6.4968	4.9836	6.0426	6.9884	5.8665	6.4473	4
1896	17	6.5539	4.2627	5.8348	6.9508			2
1898	21	6.6333	3.7612	5.6836	6.9603	4.8978	6.0379	3
1899	14	6.6294	4.2767	5.8805	7.0166	4.1109	5.8348	5
1900	14	6.5582	4.1109	6.5367	7.2406	4.5109	5.9243	4
1902	14	6.6134	4.4773	5.8972	7.0112	3.9318	5.7900	4
1903	14	6.5667	3.7136	5.2679	6.8079	5.2417	6.1549	5

1904	14	6.5309	4.3175	5.8693	6.9470	4.8122	5.9965	5
1907	1	6.6606	5.0039	6.0684	7.1009	4.8203	6.0014	4
1908	11	6.7673	4.2485	5.8608	7.1066	3.7842	5.7557	4
1909	11	6.9441	5.4596	6.2480	7.3486	5.4424	6.2226	3
1910	3	7.0536	4.0604	5.8348	7.3126	5.9814		2
1911	13	6.6333	4.0431	5.8230	7.0012	4.5326	5.8999	4
1912	12	6.8427	3.7612	5.7900	7.1420	4.5747	5.9216	4
1913	12	6.8002	4.2905	5.5334	7.0484			2
1914	12	6.9078	5.7137	6.3716	7.3683			2
1915	12	6.8189						1
1917	19	6.5930						1
1918	19	6.5667	4.5539	5.9162	6.9866	4.0943		2
1919	18	6.6307	5.3423	6.2005	7.1317	3.9703	5.7961	5
1922	20	6.5191	4.7005	6.5653	7.2356			2
1925	15	6.7742	5.3891	6.2186	7.2277	4.9904	6.0544	4
1928	2	6.7310	4.7005	5.9636	7.1123	4.0254	5.8289	4
1929	2	6.6983	4.3307	5.8777	7.0630	4.5951	5.9610	4
1931	10	6.5930	5.2983	6.2025	7.1099	3.9890	5.8171	4
1932	10	6.5971	4.7005	5.9610	7.0220	4.0073	5.8111	4
1933	6	6.6307	4.1431	5.8464	7.0067	5.2523	6.1356	4
1935	5	6.5820	4.0604	5.8081	6.9613	4.1431	5.8377	5
1937	5	6.5236	4.1897	5.8377	6.9315	4.4308	5.8889	4
1938	6	6.5425	4.9345	6.0355	7.0139	5.1240	6.0981	5
1939	5	6.5236	5.0562	6.0730	7.0166	4.4067	5.8916	3
1940	6	6.5439	4.7875	5.9814	6.9948	5.4161	6.2305	5
1941	4	6.5028	4.9558	6.0615	6.9994	4.7005		2
1942	4	6.6080	4.1109	5.8406	6.9893	5.2679	6.1485	5
1943	5	6.5667	4.8520	5.9814	7.0094	4.2047	5.8608	4
1945	17	6.6039	4.9558	6.0379	7.0536	4.3567		0
1946	17	6.6053	4.0775	5.8081	6.9773	5.1180		0
1947	21	6.6580	3.8918	5.8141	7.0157	5.2575	6.1675	3
1949	14	6.5889	4.6540	5.9610	7.0166	4.1744	5.8636	3
1952	1	6.7130	3.7377	6.3261	7.2313	5.2832	6.1759	3
1953	1	6.9295	4.5747					1
1954	3	6.9847	4.4427	5.9081	7.2779	4.6347	5.9506	4
1955	13							0
1956	12	6.8373	4.1589	5.8522	7.1546	4.3307	5.8833	4
1957	12							0
1958	1	6.6670	4.6444	5.9349	7.0596	5.1059	6.0936	4
1962	19	6.6294	4.2195					1
1963	19							0

1966	15	6.6859	4.8122	5.9865	7.0892	3.7842	5.7807	4
1967	15	7.1009	4.5539	5.9189	7.3683			2
1969	4	6.7776	4.1271	5.8319	7.1058	4.6444		2
1973	5	6.6067	4.2341	5.7869	6.9717	4.2047	5.8289	5
1974	4	6.5639	4.1109	5.8201	6.9527	4.1589	5.8348	3
1975	4	6.5236	4.3175					1
1977	5	6.5453	4.5643	5.8721	6.9575	4.6347	5.9322	5
1978	6	6.5667	4.4659					1
1979	4	6.5294	4.6913	5.9558	6.9763	5.3566	6.2066	5
1980	17	6.5944	6.1356					0
1983	21	6.9994	5.3519	6.2106	7.3740			2
1985	14							0
1986	21							0
1987	14							0
1991	11	6.7991	5.5530	6.2860	7.2682	4.9488	6.0283	3
1992	11	6.4520						1
1993	1	6.8721	4.7791	5.9915	7.2189	5.7430	6.3835	3
1994	11	6.6995	4.6634	6.4409	7.2717	4.2905	5.8665	3
1995	3	7.0012	4.6728	5.9764	7.3079	4.4998	5.9081	3
1996	1	6.7202						1
1998	11	6.7696						1
1999	12	6.8522	5.8889	6.4677	7.3715	4.3438		2
2000	12	6.7405	5.3083	6.2106	7.2034	4.2485	5.8665	3
2001	1	6.7020	4.0604	5.8171	7.0475	4.6540	5.9506	3
2003	19	6.5779	3.8067	5.7621	6.9441	5.1059	6.0890	3
2004	19	6.6464	4.9698	6.0331	7.0792	5.0938	6.0684	3
2005	19	6.6346	4.3820	5.8861	7.0220	4.6821	5.9584	4
2083	2	6.7334	4.4886	5.9081	7.0967	4.4543	5.9081	4
2087	16							0
2088	16	6.7464	4.7362	6.0014	7.1349	5.4510	6.2364	3
2089	21	6.5596	4.1897	5.8435	6.9575	4.6151	5.9558	4
2093	11	6.8711	4.2047	5.8551	7.1801			2
2095	1	6.6567	4.8442					1
2096	3	6.9801	4.5433	5.9322	7.2807	4.2341	5.8665	4
2097	13	6.6147	4.9698	6.0331	7.0588	5.0876		2
2102	19							0
2103	19							0
2104	18							0
2107	17	6.6012	4.2767	5.8435	6.9856	5.0752		0
2109	16	6.6783	4.4543	5.9243	7.0639	6.0497	6.5610	3
2110	14	6.6320	4.8903	6.0776	7.0859	4.8520	5.7683	3

2111	16	6.6451	5.4681	6.2653	7.1663			2
2113	14	6.5834	4.2195	5.8721	6.9829	3.7136	5.7961	5
2115	1	6.7393	4.5850	5.9322	7.1082	5.6595	6.3648	4
2116	1	6.6821						1
2117	1	6.7081	4.4998	5.9054	7.0783	5.6204	6.3008	4
2118	13	6.4922	5.1240	6.1026	7.0094	3.8918	5.8230	4
2119	13	6.6720	4.1744	5.8579	7.0388	4.1109	5.8171	4
2120	12	6.8265	5.2933	6.1883	7.2506	5.3279	6.2005	3
2121	12	6.8309						1
2122	8	6.7250						1
2125	18	6.5596	3.9703	5.8141	6.9479	3.5553	5.8171	5
2126	18	6.7957	4.1744	5.8693	7.1293	4.2767		0
2128	16	6.7867						1
2130	16	6.7935	4.9836					1
2131	21	6.5410	4.6151	5.9506	6.9819	4.4188		2
2132	16	6.7393	5.4596	6.2403	7.2138		5.9738	3
2133	11	6.7262	4.6540	5.9402	7.1017	5.0562	6.0730	4
2134	11	6.7639	4.0073	5.8377	7.0975			2
2135	1	6.7429	4.0943	5.8289	7.0800	5.1761	6.1334	4
2136	3	6.9976						1
2137	1	6.6995	4.6728	5.9687	7.0926	4.4773	5.9081	4
2138	13	6.5889						1
2139	13	6.5497	6.1003					1
2140	13	6.5013	5.4205	6.2344	7.0699	4.7449	5.9865	3
2141	12	6.8090	6.1570	6.6307	7.4170	5.5984		0
2148	18	6.7093	4.5539	5.9189	7.0834	4.4427	5.9108	3
2150	4							0
2152	16	6.7788	4.4543	5.9189	7.1317	5.5175		2
2153	16	6.7346	4.1271	5.8377	7.0767	5.0106	6.0615	3
2154	21							0
2155	14	6.5709	4.2905	5.8608	6.9707	4.0073		2
2156	14	6.5958						1
2159	11	6.9393	4.0775	5.8406	7.2269	4.7095		2
2161	3	6.9939	4.9273	5.9661	7.2998	4.6913	6.0064	3
2162	1	6.6464	6.0137	6.5511	7.2930	4.6913	6.0426	3
2163	1	6.7250	3.8286	5.7777	7.0527	5.1705	6.1312	4
2164	1	6.7382	5.4681	6.2953	7.2342	4.6052		2
2165	3	6.9967	4.2767	5.8693	7.2772			2
2166	13	6.6134	4.7185	5.9480	7.0282	5.0876	5.7777	3
2167	13	6.5723	6.0615					1
2168	12	6.6995	5.1874	6.1356	7.1499	4.3820	5.9108	4

2169	1	6.6554	3.9120	5.7777	7.0031	5.1761	6.0753	4
2173	19	6.6758	3.8712	5.7683	7.0148			2
2174	18	6.8997	5.4293	6.3491	7.3550		5.7869	4
2175	18	6.6682	5.4806	6.2653	7.1801	4.8203	6.0088	3
2177	4	6.5610	3.9703	5.8021	6.9451			2
2178	21	6.6093	5.4972	6.2519	7.1397	4.9127	6.0137	3
2179	16	6.7856	5.5013	6.4785	7.3369	5.3982		0
2180	16	6.6946	4.2627	5.8693	7.0579	4.3438	5.8693	4
2184	1	6.6746						1
2185	1	6.7105	5.3230	6.1862	7.1755	5.3083	6.1675	3
2186	13	6.6720	4.3307	5.8721	7.0432	4.0073	5.8081	4
2187	13	6.5653	4.7005	6.4345	7.1952			2
2188	13	6.5944	4.5747	5.9402	7.0130	4.6821	5.9636	3
2189	13	6.5468						1
2190	1	6.7154	4.3944	5.9026	7.0825	3.8712	5.7869	3
2191	12	6.7685	4.7536	6.2226	7.2255	4.0604	5.8406	4
2194	8	6.7754	5.9890	6.5043	7.3421			2
2195	19	6.6214	4.2341	5.8693	7.0076	4.1897	5.8435	5
2196	18	6.6120	5.8805	6.4583	7.2313			2
2198	20	6.5147	5.0239	6.0730	7.0112	5.0562	6.0684	3
2200	14	6.8057						1
2201	21	6.6386	4.6052	5.9454	7.0440	4.3438	5.8944	4
2202	16							0
2205	11	6.6983						1
2206	1	6.6884	5.0999	6.1092	7.1333			2
2207	1	6.6503	5.8944	6.5453	7.2923	5.7589		2
2209	3	6.9958						1
2210	13	6.5309	5.0370	6.0661	7.0184	4.2047	5.7807	4
2211	13	6.6147	5.2417	6.1420	7.0992	4.5109	5.9269	3
2212	13	6.6995	4.8752					1
2213	13	6.6884	4.7095	5.9636	7.0834	4.9904	6.0497	4
2214	12	6.7190	4.0943	5.8319	7.0639	4.9628	6.0426	4
2215	12	6.7867						1
2219	19	6.6227	4.3438	5.8377	6.9985			2
2220	18	6.6438	4.0943	5.8636	7.0211	4.6444	5.9349	4
2221	18	6.6120	4.2767	5.8608	6.9985	4.8363	6.0331	3
2222	18	6.6067	4.2627	5.8861	7.0031	4.6347	5.9789	3
2224	4	6.5396	3.9512	5.8051	6.9315	4.8903	6.0210	5
2225	5	6.5309	4.0254	5.8141	6.9285			2
2227	14							0
2228	14	6.6053						1

2229	14	6.5737						1
2232	11	6.7901	3.9318	5.8171	7.1107			2
2233	1	6.7007	5.2204	6.1506	7.1562	3.9120		2
2234	3	6.9930	4.7791					1
2235	13	6.5439	4.6728					1
2236	13	6.6147	5.1705	6.1181	7.0901	4.6444	5.9402	4
2237	12	6.7776	5.0938	6.1225	7.1959	4.4308	5.9081	3
2239	8	6.7238	6.4409	6.8046	7.4582			2
2241	18	6.5751	4.3567	5.8944	6.9847	3.9703	5.8171	3
2242	18	6.5667	5.3230	6.1506	7.0733	4.0431	5.8348	4
2250	13	6.4739	4.9488	6.0426	6.9745			2
2251	20	6.5367	4.3694	5.8805	6.9546	4.4308		2

#L1, lactation 1; L2, lactation 2; AFC, age at first calving; ASC, age at second calving, DC, days from calving to conception; CI, calving interval. Log_e values were used to normalise distributions, except for the number of calvings.
