# Supplementary material for: Polymorphisms in the selectin gene cluster are associated with fertility and survival time in a population of Holstein Friesian cows
Source: PLoS One. 2017 Apr 18;12(4):e0175555. doi: 10.1371/journal.pone.0175555 (PMC5395145; doi:10.1371/journal.pone.0175555)
Supplement: S4 Table — (PDF) [file pone.0175555.s004.pdf]

S4 Table. Survival times for the cows included in the study.

Cow ID	Farm ID	Culled<br>0 = No<br>1=Yes	Days birth to culling#
607	11	1	1688
845	17	0	2340
847	17	0	2340
848	17	0	2340
849	3	1	234
850	17	0	2340
851	3	0	2340
852	20	0	2340
1342	5	1	843
1343	3	1	230
1344	3	0	2340
1345	18	1	1491
1347	21	0	2340
1348	11	1	906
1349	3	1	237
1350	21	1	922
1352	18	0	2340
1353	5	1	928
1354	6	1	2096
1355	3	0	2340
1356	6	1	1970
1357	21	1	376
1360	6	1	2144
1361	21	0	2340
1362	3	1	1972
1363	3	1	1905
1699	15	1	1633
1701	15	0	2340
1702	2	1	1253
1703	2	1	1967
1704	2	1	800
1706	10	0	2340
1709	4	1	1085
1710	6	1	857
1712	4	1	1187
1714	6	1	1558
1715	6	1	2014
1717	6	1	1379
1720	16	0	2340
1723	21	1	1226
1725	16	1	1263
1726	16	1	1879

1727	14	1	1640
1729	9	1	1090
1731	1	0	2340
1732	11	0	2340
1733	1	1	1704
1734	1	1	1502
1735	11	0	2340
1736	13	0	2340
1738	19	1	1697
1739	19	1	1475
1740	18	0	2340
1744	20	1	1511
1745	20	1	1486
1746	15	0	2340
1748	15	0	2340
1750	2	1	1306
1751	2	0	2340
1753	6	1	2066
1755	5	1	954
1757	4	1	318
1758	4	1	851
1760	4	1	2332
1761	5	1	1393
1762	6	1	1495
1763	6	1	1791
1764	6	1	1972
1765	5	0	2340
1766	6	0	2340
1767	17	1	1244
1768	17	0	2340
1770	16	1	1796
1772	21	0	2340
1773	21	0	2340
1776	14	0	2340
1777	14	1	1918
1781	11	1	1262
1782	3	1	1476
1783	1	1	1310
1785	11	0	2340
1786	3	0	2340
1787	12	0	2340
1789	19	1	1490
1790	19	1	1948
1791	19	1	1693
1792	19	1	1470
1796	15	1	978

1798	15	1	2193
1799	2	0	2340
1800	2	1	1367
1801	2	0	2340
1802	10	0	2340
1804	5	1	1860
1810	4	1	1654
1811	5	1	2207
1812	4	1	2035
1813	5	0	2340
1816	16	1	857
1817	16	0	2340
1819	14	1	1603
1820	14	1	1566
1821	14	1	1825
1822	14	1	962
1824	11	1	1578
1825	1	0	2340
1826	1	0	2340
1827	3	0	2340
1828	13	1	1809
1829	12	1	1353
1830	19	1	1757
1831	19	1	1120
1832	19	0	2340
1834	18	1	1557
1835	15	0	2340
1837	15	1	2008
1838	2	1	2292
1839	10	0	2340
1840	5	1	1646
1841	10	0	2340
1843	6	0	2340
1845	5	1	850
1846	6	1	1438
1847	4	1	2004
1848	5	1	2270
1849	4	1	1157
1850	4	1	1493
1852	16	1	1949
1853	21	0	2340
1854	21	1	921
1855	14	0	2340
1857	14	0	2340
1861	3	1	264
1862	3	0	2340

1863	3	0	2340
1864	13	0	2340
1865	13	1	1523
1866	3	0	2340
1867	13	0	2340
1868	13	1	1935
1869	12	1	1001
1870	19	1	1104
1871	19	1	1982
1874	18	1	971
1875	18	0	2340
1879	2	0	2340
1880	2	1	1866
1881	2	1	1782
1883	10	0	2340
1884	6	1	1390
1885	4	1	1162
1889	6	0	2340
1890	4	1	1772
1891	4	1	1724
1893	4	0	2340
1896	17	1	1044
1898	21	0	2340
1899	14	0	2340
1900	14	0	2340
1902	14	1	1939
1903	14	0	2340
1904	14	0	2340
1907	1	0	2340
1908	11	0	2340
1909	11	0	2340
1910	3	1	2111
1911	13	0	2340
1912	12	0	2340
1913	12	1	1153
1914	12	1	1658
1915	12	1	933
1917	19	1	1484
1918	19	1	1690
1919	18	0	2340
1922	20	1	1409
1925	15	0	2340
1928	2	1	2257
1929	2	0	2340
1931	10	0	2340
1932	10	0	2340

1933	6	0	2340
1935	5	0	2340
1937	5	1	2072
1938	6	0	2340
1939	5	1	1877
1940	6	0	2340
1941	4	1	1530
1942	4	0	2340
1943	5	1	2058
1945	17	0	2340
1946	17	0	2340
1947	21	0	2340
1949	14	1	1557
1952	1	0	2340
1953	1	1	1301
1954	3	0	2340
1955	13	1	885
1956	12	0	2340
1957	12	1	1861
1958	1	0	2340
1962	19	1	1619
1963	19	1	548
1966	15	0	2340
1967	15	1	1813
1969	4	1	1575
1973	5	1	2089
1974	4	1	1398
1975	4	1	960
1977	5	0	2340
1978	6	1	1149
1979	4	0	2340
1980	17	0	2340
1983	21	1	1884
1985	14	1	335
1986	21	1	906
1987	14	1	321
1991	11	0	2340
1992	11	1	645
1993	1	0	2340
1994	11	0	2340
1995	3	1	2202
1996	1	1	1028
1998	11	1	1331
1999	12	1	1845
2000	12	0	2340
2001	1	1	2036

2003	19	1	1709
2004	19	1	1658
2005	19	0	2340
2083	2	0	2340
2087	16	1	1141
2088	16	0	2340
2089	21	0	2340
2093	11	1	1478
2095	1	1	1534
2096	3	0	2340
2097	13	1	1555
2102	19	1	954
2103	19	1	832
2104	18	1	799
2107	17	0	2340
2109	16	0	2340
2110	14	1	1675
2111	16	1	1565
2113	14	0	2340
2115	1	0	2340
2116	1	1	838
2117	1	0	2340
2118	13	0	2340
2119	13	0	2340
2120	12	0	2340
2121	12	1	961
2122	8	1	855
2125	18	0	2340
2126	18	0	2340
2128	16	1	1305
2130	16	1	1418
2131	21	1	1485
2132	16	0	2340
2133	11	0	2340
2134	11	1	1810
2135	1	0	2340
2136	3	1	1561
2137	1	0	2340
2138	13	1	730
2139	13	1	1235
2140	13	0	2340
2141	12	0	2340
2148	18	0	2340
2150	4	1	847
2152	16	1	1727
2153	16	0	2340

2154	21	1	910
2155	14	1	1384
2156	14	1	903
2159	11	1	1713
2161	3	0	2340
2162	1	1	1988
2163	1	0	2340
2164	1	1	1614
2165	3	1	1552
2166	13	1	1545
2167	13	1	1195
2168	12	0	2340
2169	1	1	1932
2173	19	1	1481
2174	18	0	2340
2175	18	0	2340
2177	4	1	1113
2178	21	0	2340
2179	16	0	2340
2180	16	0	2340
2184	1	1	849
2185	1	1	2129
2186	13	0	2340
2187	13	1	1459
2188	13	1	1965
2189	13	1	830
2190	1	1	1686
2191	12	0	2340
2194	8	1	1830
2195	19	0	2340
2196	18	1	1524
2198	20	0	2340
2200	14	1	980
2201	21	0	2340
2202	16	1	1251
2205	11	1	1619
2206	1	1	1381
2207	1	1	2033
2209	3	1	1275
2210	13	0	2340
2211	13	1	1643
2212	13	1	1117
2213	13	0	2340
2214	12	0	2340
2215	12	1	919
2219	19	1	1098

2220	18	0	2340
2221	18	0	2340
2222	18	1	1574
2224	4	0	2340
2225	5	1	1422
2227	14	1	800
2228	14	1	1052
2229	14	1	951
2232	11	1	1248
2233	1	1	1603
2234	3	1	1359
2235	13	1	844
2236	13	0	2340
2237	12	0	2340
2239	8	1	1735
2241	18	0	2340
2242	18	0	2340
2250	13	1	1554
2251	20	1	1206

Censored at 2340 days of age
