# Supplementary material for: Polymorphisms in the selectin gene cluster are associated with fertility and survival time in a population of Holstein Friesian cows
Source: PLoS One. 2017 Apr 18;12(4):e0175555. doi: 10.1371/journal.pone.0175555 (PMC5395145; doi:10.1371/journal.pone.0175555)
Supplement: S5 Table — (PDF) [file pone.0175555.s005.pdf]

S5 Table. Milk phenotypes for the cows included in the study#.

Cow ID	Farm ID	TMY_L1	305d_L1	PMY_L1	TMY_L2	305d_L2	PMY_L2	SCC class
607	11	10920	10516	43.4	10459		57.4	0
845	17	7604	7384		10206	9905		
847	17	8271	7581		9377			
848	17	5654			7893			
849	3							
850	17	7649			9497			
851	3	6415	6063	31.2	6860		40.1	0
852	20	8744	7060	37.1	9055	8207	40.5	
1342	5							
1343	3							
1344	3	8314		35.5	12185	11324		1
1345	18	12848	7211	29.8				1
1347	21	7328		35.6	8509		39.6	0
1348	11	2689		28.3				
1349	3							
1350	21							
1352	18	8695	8572	39.2	11776	11103	45.6	1
1353	5	4344		37.1				
1354	6	11991	10140	35.9	13855	12052	44.1	
1355	3	8217	6942	31.9	9338		46.3	2
1356	6	10264		37.4	9496	9065	40.1	2
1357	21							
1360	6	7661		29	5549		22.2	1
1361	21	7898		34.8	7754		40	1
1362	3	6454	6341	25.5	7642	7465	32.1	2
1363	3	7485		30.3	8456		43.8	1
1699	15	6099	6036	24.9	7532	7135	33.4	
1701	15	5866	5641	26.8	8376		34.8	
1702	2	435						
1703	2	14943	8146	34.3	11448	11086		1
1704	2							
1706	10	14449	7686		11340	9880		2
1709	4	7526		25.5	197			
1710	6							
1712	4	7389		32.3	3814		15.9	
1714	6	8857	8857	29.3	14953	10101	39.2	0
1715	6	9742	9564	35.4	10388	9622	38	1
1717	6	8076	7203	29.7				

1720	16	8684		37.5	12229	10626	45.5	1
1723	21	8483	8154	37.2				1
1725	16	12316	11041	41.7				
1726	16	11141	9939	42	7338	4120	22	1
1727	14	8411		33.7	8914	8588	38.9	1
1729	9							
1731	1	10285	9271	36	9919		43	1
1732	11	11751	11115	44.5			47.4	2
1733	1	8122		32.3	11631	11357	45.5	1
1734	1	13112	8445	35.4				0
1735	11	5256	5256	25.7	10262	10032	45.6	1
1736	13	8899	8086	34.4	11922			1
1738	19	14077	10600		7128	7027	39.8	1
1739	19	11788	11079		9931	9370	38.3	0
1740	18	9422		39.1	11993	11888	48.2	2
1744	20	10046	8753	35.6	7283		32.4	
1745	20	7899	7682		9373		38.6	
1746	15	6644		27.4	9340	8728	34.5	
1748	15	9114	8418	37.2	8246	7928	41	
1750	2	8526	7735	30.9	4131		42.7	0
1751	2	129			8806	8122	34	2
1753	6	9071	8317	28.2	12678	10970	52.7	2
1755	5	2408		26.1				
1757	4							
1758	4							
1760	4	12338	8366	31.3	13816	10801	49.2	
1761	5	9021	7490	29.5	5014		42.5	
1762	6	10064	8976	37.6	9207	9207	41	
1763	6	12723	8322	30.6	13413	11287	56.1	
1764	6	8357	8130	28.2	8764	7687	42.5	1
1765	5	7337	7337	29.4	10818	9536	43.7	
1766	6	10776	8969	35.6	9048	8447	34.8	
1767	17	1479						
1768	17	11690	6739					
1770	16	18458	7344	28.7				1
1772	21	10572		46.2	11165		45.4	1
1773	21	8381	8346	39	9912		44	1
1776	14	8802	7862	33.9	11494	10624	42.3	1
1777	14	9973	8799	36	13233	10476	45.4	2
1781	11	9966	8640	36.8				1
1782	3	8309	8012	34				2

1783	1	12354	8923	32.3				1
1785	11	9183		44.2	11172	10253	47.7	1
1786	3							
1787	12	13370	9183	33.8	9883	9258	38.3	1
1789	19	19810	9246	32.9				0
1790	19	8786			12266	9612	36.5	0
1791	19	11820	10913		14685	12773	51.6	0
1792	19	10652	9415		5252		31.3	0
1796	15	2000						
1798	15	8212	8212	36.1	12142	9923	44	
1799	2	10866	7743	35.5	12989	10779		1
1800	2	7835	7835	29.2	5639		39.2	1
1801	2	8714	8400		10756	9150	36.1	0
1802	10	8532	7757		9291			1
1804	5	10736	8267	33	11026	8918	35	1
1810	4	10919	9738	33	7509	7509	31.6	
1811	5	10291	9348	37.9	12173	11059	43.9	
1812	4	9504		39.9	14374	12933	54.7	1
1813	5	11226	9880	38.4	10526	10320	42.6	
1816	16	1526						
1817	16	7449		32.2	8446		39.4	2
1819	14	7641		31.1	11225	10314	46.3	1
1820	14	12069	9358	36.5	6379		43.3	0
1821	14	7401	7085	28.3	9200	9200	45	1
1822	14	4404		19				0
1824	11	9948	9036	38.7	9640		54.3	2
1825	1	9606		37.9	11551		44.2	2
1826	1	12782	10404	37.4	16530	11867	45.8	1
1827	3	8126	7774	34.6	12362	9920	43.6	2
1828	13	8733	7671	27.4	11487		42.8	0
1829	12	10387	7575	30				1
1830	19	28500	10096	41.7				0
1831	19	8263			2994			
1832	19	10555			14139	12426	50.8	0
1834	18	17022	7179	29.2				0
1835	15	8741		36.1	10649	10385	41	
1837	15	12061	8636	34.5	7912		37	
1838	2	7508	7117	32.7	7151		34.4	1
1839	10	9816	8369		12505	9163		2
1840	5	10082	8657	30.6	10962	10042	42.8	1
1841	10	9949	8889		10155	10059		1

1843	6	8384	8332	27.4	7603	7603	30.7	
1845	5							
1846	6	12678	11033	40	9411	8467	41.1	1
1847	4	9912	9827	35.8	10926	9656	42.8	1
1848	5	1179		11.2	12818	11282	39.3	1
1849	4	9418	8773	40.3				
1850	4	8455		32.3	10137	9057	35.1	
1852	16	10953	10042	42	14694	9370	39.6	1
1853	21	8507	8250	36.6		9807	39.4	
1854	21							
1855	14	13897	8185	33.2	10841	10256	43.4	1
1857	14	6982		29.7	9312		46	2
1861	3							
1862	3	6493	6093	24.5	7419		35.9	1
1863	3	8196		32.4	9595		42	2
1864	13	13253	11140	40.4	11678	11486	48.1	1
1865	13	12764	10513	42.5	6670		33.1	0
1866	3	9730	8381	33.6	10750	10669	50	1
1867	13	8814	8623	37.2	8385		49.8	2
1868	13	13327	11756	43	20817		53.4	0
1869	12	3253		30.7				
1870	19	2896		25.8				
1871	19	12699	10630		15040	12985	60.2	0
1874	18	4991		38.5				
1875	18	6391	6459	33.9	9495	9624	41.9	1
1879	2	7855	6595	31.6	10714	9597	46.6	1
1880	2	8539	8062	35.4	10424	8845	35.7	1
1881	2	10631	9669	38.5				0
1883	10	8194			6846	6364		0
1884	6	9902	8827	30.7	7490	7313	34	1
1885	4	14908	11040	37				
1889	6	10516	8875	34.7	11123		48	1
1890	4	7822	7480	23.8	8733	8658	38.1	2
1891	4	10412	10412	33.3	10896	8899	40.2	
1893	4	6543	5472	23.4	9427	8291	32.6	1
1896	17	3954						
1898	21	7786		36	7682	7329	29	0
1899	14	7778		33.7	10057	10057	49.4	1
1900	14	14345	8238	37.7	9730		39.7	0
1902	14	7730	7485	30.4	10593		47.7	1
1903	14	4263		25.6	7965	6109	23.2	2

1904	14	7476		35.3	8034	7720	38.6	1
1907	1	11305	9804		13647	12405	47.9	0
1908	11	3549		43.2	7683		42.2	1
1909	11	16241	11175	41.6	16381	12736	55.8	
1910	3	7461		32.6	17554	9246	38.1	2
1911	13	6731		26	8306	8070	32.8	1
1912	12	7411		35.2	7228	6965	33.8	1
1913	12	6627		29.3				0
1914	12	14113	10089	40.5	1797			0
1915	12	427						
1917	19	20864	10570	43.7				0
1918	19	9210	8698		14866	10532	48.9	1
1919	18	13500	10353	37.9	10053		46	1
1922	20	12727	8770	35.3	531			
1925	15	10322	7767	32.1	11873	10770	46	
1928	2	8311	7617	33	8320		48	1
1929	2	7883	7883	37.3	10426	10135	48.6	1
1931	10	11964	8850		8114			1
1932	10	7886	7510		7935			1
1933	6	9426		34.2	12287	10100	45.5	
1935	5	8527		29.1	7883	7883	33.1	1
1937	5	8006	8006	28.7	7640	7433	33.5	
1938	6	8911	7700	31.5	10879	9568	37.8	1
1939	5	11553	9379	37.4	9378	8935	36	1
1940	6	10540	9294	33.8	10120	9048	35.8	
1941	4	12998	10781	37.4	13918	11391	43.8	2
1942	4	9242		33.6	12290	10900	40.5	
1943	5	8631	7677	36.4	9152	9152	33.2	
1945	17	4623			9107	9107		
1946	17	6277			11606	9569		
1947	21	5229		26.2	8463	7292	33.8	1
1949	14	7634	6898	26.9	9834	9771	38.3	2
1952	1	12399	8894	35.7	13427	10863	50	0
1953	1	6062		28.5				2
1954	3	7603		29.4	8543	8364	39.6	2
1955	13							
1956	12	8442	8382	34.2	9546	9443	48.9	1
1957	12							
1958	1	9996	9422	33.9	14034	12047	46	2
1962	19	19831	10139	36.4				0
1963	19							

1966	15	9159	8476	33.2	7890		41.3	
1967	15	8334	8073	33	6588		39.6	
1969	4	7895		36.6	10067	10025	58.6	
1973	5	8497		29.4	11179	11179	43.1	1
1974	4	5161		20.5	5141		21.6	
1975	4	9287		34.4				
1977	5	9438	9438	33	9462	8596	36.4	
1978	6	7713		28.1				
1979	4	10223	9227	31.2	11328	10356		1
1980	17	15677	7849					
1983	21	7345	6522	28.6	8032		49.4	2
1985	14							
1986	21							
1987	14							
1991	11	16245	10958	48.7	15126	12852	46.7	1
1992	11							
1993	1	11755	10682	39.8	17775	11816	47.1	0
1994	11	13763	8273	40.7	11702	11533	46.8	2
1995	3	5862	5645	21.4	6843	6754	31.3	2
1996	1	3533		24.9				0
1998	11	9344	6792	38.2				2
1999	12	12111	7676	33.4	6859		34.1	2
2000	12	11414	8027	34.7	10092	10092	43.2	1
2001	1	7701		36.2	9456	9267	43	1
2003	19	7897			13295	11299	45.7	0
2004	19	13290	10529		16869	14184	56.5	0
2005	19	11160	10816		12618	12414	48.9	0
2083	2	12683	11456	48.2	13993	13993		2
2087	16							
2088	16	7844	7395	28.8	12763	10376	45.6	1
2089	21	7225		31.8	8725		40	1
2093	11	10907		47.5	5840		47.5	2
2095	1	12342	7812	29.3				0
2096	3	5720	5555	23.5	6858		31.7	2
2097	13	9560	8760	35.8	9999			0
2102	19							
2103	19							
2104	18							
2107	17	6728			11000	9859		
2109	16	10145	10011	39.2	20230	13283	52.1	1
2110	14	8515	7455	30.4	11622	11048	46.6	2

2111	16	12691	8569	32		8700	41.5	1
2113	14	7471		37.7	8335		37.4	1
2115	1	7732	7571		13040	11212	47.6	0
2116	1	1048		26.2				
2117	1	7843	7802	34	13508	10655	39.8	0
2118	13	12234	9583	35	12126	9357	49.4	1
2119	13	8912			10224			2
2120	12	10666	7386	37.2	12211	9461	40	1
2121	12	588		16.2				
2122	8	232						
2125	18	8501	8652	35.9	9356	9413	42.4	0
2126	18	11761	11416	41.8		11420	45.4	2
2128	16	10672	8676					
2130	16	16436	11187	49.9				2
2131	21	6351	6261	23.6	8097	7038	32.2	2
2132	16	13280	10329	43.2	11350	9765	42.4	1
2133	11	8040	7397	31.5	12517	10820	45.5	2
2134	11	7052		34.5	13716	10386	45.6	1
2135	1	9840		37.9	13956	11862	46.4	1
2136	3	8226	7339	27.1				1
2137	1	10727	10204	39.5	10454	10394	39.1	1
2138	13	118						
2139	13	13652	8753	31.2				0
2140	13	12262	8709	35.4	11734			2
2141	12	18280	9152	36.6	13424	10373	43.5	2
2148	18	7944	7512	30.6	10289	9635	41.9	0
2150	4							
2152	16	6846	6758	27	6673		29.1	1
2153	16	6990		29.5	11039	10050	41.1	2
2154	21							
2155	14	8303		32.8	10026		41.8	0
2156	14	1188						
2159	11	5517		25.3	6668		34	
2161	3	8079	7486	27.6	5302		24.4	1
2162	1	9674	6677	26	15171	12677	58.1	0
2163	1	8357		34.2	13720	11816	47.1	2
2164	1	12016	8678	32.9	7943		42.4	1
2165	3	8759		37.5	4250		42.4	2
2166	13	10795	10020	36	10235	9860		1
2167	13	11950	8810	33.8				2
2168	12	7356	5830	23.4	8801	8629	41.5	1

2169	1	6908		29.6	12477	10994	43.8	2
2173	19	4448			8385	7894	44.8	1
2174	18	18043	12561	46.8		11334		2
2175	18	13867	9798	35		12603	49.1	1
2177	4	9214		32.5	2896			
2178	21	8973	8169	31.6	13024	10225	36	1
2179	16	17786	11262	42.5		10924	44.2	1
2180	16	9862		39.9	11523	11523	46.5	1
2184	1	1541		29.4				
2185	1	12913	9825	37.7	15544	12441	47.3	1
2186	13	10136		39.4	9684		42.2	1
2187	13	12388	11322	41.5	3593			0
2188	13	12254	12136	43.8	13347	12914	49.9	1
2189	13	2738						
2190	1	8155		29.7	9585		42.5	0
2191	12	10747	8664	35.3	7955		43	2
2194	8	17699	11224		9945			1
2195	19	9792			12330	12330		
2196	18	16513	11182	46.1	7358		56.8	1
2198	20	5681	4905	23.4	10494	9579	40.6	
2200	14	766		23.4				
2201	21	7378	7339	31.8	9523	9680	40.6	1
2202	16							
2205	11	22721	11391	60				0
2206	1	13080	10375		5937		51.5	2
2207	1	16859	9573	34.2	17088	12039	50.7	0
2209	3	3482		30.3				
2210	13	11624	9310	36.9	11190		47.5	1
2211	13	12542	10572	40.4	10059			2
2212	13	10985		40.2				0
2213	13	9281	8765		13926			2
2214	12	7356	7168	37.3	9301	8033	37.3	1
2215	12	523						
2219	19	9233	9210					
2220	18	8947		34.5	12247	11497	48.5	1
2221	18	5560		27.8	8890	8111	31.6	0
2222	18	9138	9138	35.7	12298	11561	54.1	0
2224	4	8016		29.5	11438	10011	39.7	1
2225	5	10899	10899	37.8	11756	9453	35.2	1
2227	14							
2228	14	7790	7609	30.6				1

2229	14	3475		19.5				
2232	11	7031		37.9	742			2
2233	1	8620	6823	25	10985	10722	42.6	2
2234	3	3143		14.3				1
2235	13	5413						
2236	13	11657	9534	34.5	12232			2
2237	12	13524	10853	46.9	10258	10110	46.2	1
2239	8	23856	11456					0
2241	18	7588	7423	34	9242		40.1	0
2242	18	16972	12527	49	12132		49.8	1
2250	13	10511	8706	34.2	4557			
2251	20	7209		28	4432		28	

#L1, lactation 1; L2, lactation 2; TMY, total milk yield; 305d, 305-day milk yield; PY, peak milk yield. SCC class. Animals were classified as affected, intermediate or unaffected animals. Unaffected animals never had a SCC >200,000 cells mL^{-1} (class 0). Affected cows, classified as mastitic, had a SCC >300,000 cells mL^{-1} in two consecutive tests or in four non-consecutive tests, or were cows with a SCC >500,000 cells mL^{-1} in any one test during any lactation period, regardless of parity (class 2). Intermediate animals were placed in class 1.
